# Synthesis of constrained analogues of tryptophan

**DOI:** 10.3762/bjoc.11.216

**Published:** 2015-10-27

**Authors:** Elisabetta Rossi, Valentina Pirovano, Marco Negrato, Giorgio Abbiati, Monica Dell’Acqua

**Affiliations:** 1 Dipartimento di Scienze Farmaceutiche, Sezione di Chimica Generale e Organica “A. Marchesini”, Università degli Studi di Milano, Via Venezian, 21, 20133 Milano, Italy

**Keywords:** constrained tryptophans, Diels–Alder, indoles, tetrahydrocarbazoles, unnatural amino acids

## Abstract

A Lewis acid-catalysed diastereoselective [4 + 2] cycloaddition of vinylindoles and methyl 2-acetamidoacrylate, leading to methyl 3-acetamido-1,2,3,4-tetrahydrocarbazole-3-carboxylate derivatives, is described. Treatment of the obtained cycloadducts under hydrolytic conditions results in the preparation of a small library of compounds bearing the free amino acid function at C-3 and pertaining to the class of constrained tryptophan analogues.

## Introduction

**Figure 1:**
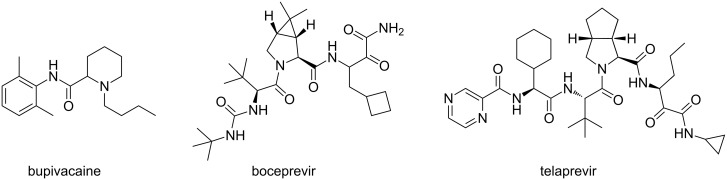
Examples of drugs embodying unnatural amino acids.

With the term of “unnatural” amino acids, a plethora of naturally occurring or chemically synthesized non-proteinogenic amino acids are classified [1]. Chemically synthesized non-proteinogenic amino acids embody in principle a countless collection of assorted chemical structures and are mainly employed as they are or as scaffolds for pharmacologically active products and biochemical studies. In recent years, both pharmaceutical companies and academics became interested in the design and synthesis of peptidomimetics and peptide analogues as new therapeutic drugs [2–3]. Medicinal chemistry progress in these fields was probably inspired by the biochemical advancements in the recognition of new naturally occurring peptides possessing useful biological activities and in the elucidation of their physiological functions. However, peptides assembled with natural amino acids present several drawbacks related to metabolic instability, deficiency in selective interactions and reduced oral absorption that prevent their use in therapy [4]. On the other hand, peptidomimetics offer the advantage of nearly countless manipulations in order to control the biological functions, stability, potency, and ADME parameters [5]. In particular, the inclusion of the amino acidic framework in a cyclic or bicyclic structure confers specific features to the synthesized molecules: well-defined secondary structure, structural rigidity, enhanced binding activity and selectivity [6–7]. For example, bupivacaine (Figure 1), commercialized by Sanofi, is a local anesthetic drug containing a six-membered ring [8]. Moreover, fused bicyclic unnatural amino acids are present in the structures of two antiviral drugs, boceprevir (Merck) [9] and telaprevir (Vertex, Johnson & Johnson) [10] used against hepatitis C genotype 1 viral infections (Figure 1).

In this research field, tryptophan analogues received less attention with respect to others congeners. Constraints and modifications in the tryptophan core have been mainly attained following two different strategies: embodying the nitrogen atom of the amino acid function in a β-carboline framework or inserting a linking group between the α-carbon atom of the amino acid function and the C-2 carbon of the indole ring (tetrahydrocarbazole derivatives).

**Figure 2:**
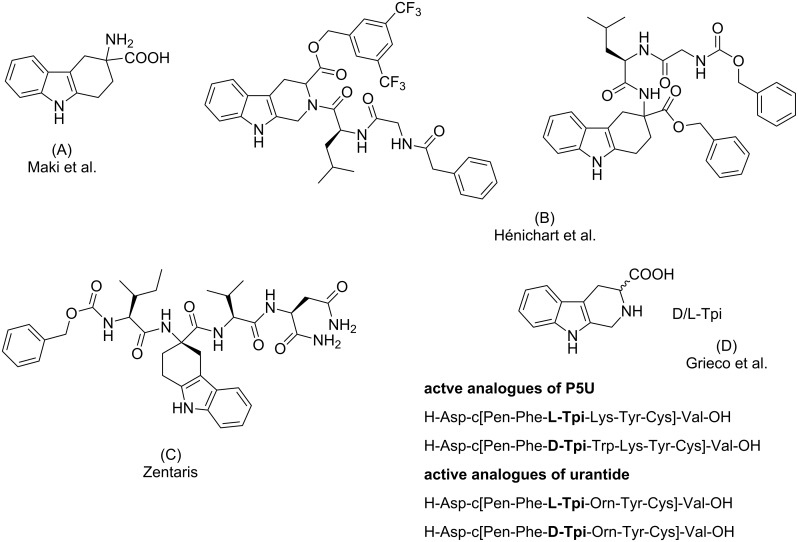
Examples of biologically active compounds embodying constrained analogues of tryptophan.

The first report on the synthesis and biological evaluation of a constrained tryptophan analogue appeared in the literature in 1973 [11]. Maki and co-workers reported the synthesis of 3-amino-1,2,3,4-tetrahydrocarbazole-3-carboxylic acid as a rigid analogue of α-methyltryptophan, a well known unnatural amino acid able to inhibit α-chymotrypsin activity, Figure 2 (A). Hardening tryptophan in β-carboline or carbazole frameworks has been used by Hénichart and co-workers in their studies devoted to the identification of new dual NK1/NK2 antagonists [12], as shown in Figure 2 (B).

In 2003, the pharmaceutical company Zentaris patented a series of tetrahydrocarbazole derivatives as ligands for G-protein-coupled receptors (GPCR), and in particular as antagonists of the gonadotropin-releasing hormone (GnRH) [13–14], Figure 2 (C). Finally, in the field of therapeutic peptides, constrained tryptophan residues of the β-carboline family (L-Tpi and D-Tpi), were used by Grieco and co-workers in the study and development of urotensin-II receptor (UTR) peptide ligands [15], Figure 2 (D).

Switching from the biological evaluation to the chemistry of tryptophan analogues, the above described two categories were synthesized according to the Pictet–Spengler reaction or by Fischer indole synthesis [11–14]. However, Fischer indolization suffers from the lack of regioselectivity depending on the substitution pattern of starting materials (substituted arylhydrazines and cyclohexanones) and is therefore useful mainly for the synthesis of symmetrically or unsubstituted derivatives [16–17].

**Scheme 1:**
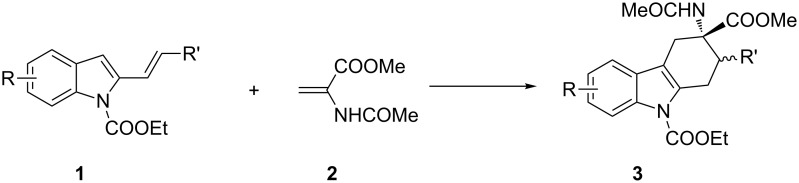
Planned Diels–Alder reactions for the synthesis of tetrahydrocarbazoles as constrained analogues of tryptophan.

Recently, our research group described the synthesis of tetrahydrocarbazole derivatives by Diels–Alder reactions between 2-vinylindoles as 4π-components with activated dienophiles and allenes [18–21]. The reported methodologies allowed for the synthesis of substituted derivatives with excellent degrees of selectivity. Starting from these results, we envisaged that 3-amino-1,2,3,4-tetrahydrocarbazole-3-carboxylic acid derivatives **3**, constrained analogues of tryptophan, could be synthesized by Diels–Alder reactions between 2-vinylindoles **1** [22] as dienes and methyl 2-acetamidoacrylate (dehydroalanine) **2** [23] as dienophile (Scheme 1).

Reported [4 + 2] cycloaddition reactions of methyl 2-acetamidoacrylate (**2**) and its congeners with cyclic/acyclic dienes and azadienes occur under conventional heating or microwave irradiation [24]. Moreover, the use of titanium tetrachloride as Lewis acidic promoter has been reported [25]. Finally, simple functionalization reactions of indoles with **2** are reported in the literature [26–29].

In this paper we report our findings about the reactions between 2-vinylindoles **1** and methyl 2-acetamidoacrylate (**2**) resulting in the synthesis of a small library of 3-amino-1,2,3,4-tetrahydrocarbazole-3-carboxylic acid derivatives **3**. Moreover, a study was focused on the complete deprotection of the cycloadducts in order to obtain the free amino acid function.

## Results

2-Vinylindole **1a** was selected as a benchmark substrate to evaluate the feasibility of the devised [4 + 2] cycloaddition reaction with methyl 2-acetamidoacrylate (**2**). Test conditions and obtained results are summarized in Table 1.

**Table 1:** Screening of reaction conditions for the cycloaddition reaction between **1a** and **2**.^a^

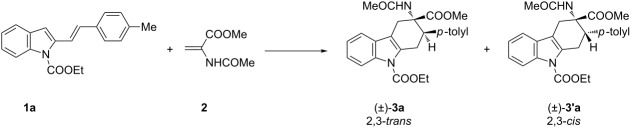							

Entry	Catalyst	mol %	Solvent	*T*, °C	Time, h	Yield, %	dr **3a**:**3’a**

1	–	–	toluene	110	24	NR	–
2	Mg(ClO_4_)_2_	15	toluene	110	24	NR^b^	–
3	Sc(OTf)_3_	15	toluene	110	24	NR^b^	–
4	Sc(OTf)_3_	15	CHCl_3_	40	24	NR^b^	–
5	Cu(OTf)_2_	30	CHCl_3_	60	24	NR^c^	–
6	BF_3_·OEt_2_	15	toluene	rt, 100	48	NR^b^	–
7	BF_3_·OEt_2_	50	toluene	rt, 100	48	NR^b^	–
8	AuCl_3_	5	toluene	rt, 100	24	NR^b^	–
9	Au(PPh_3_)Cl/AgOTf	2	toluene	rt, 100	22	20^b^	1:1
10	EtAlCl_2_	20	CHCl_3_	rt	48	35^b^	>98:2
11	EtAlCl_2_	20	toluene	rt	48	30^b^	>98:2
12	EtAlCl_2_	100	CHCl_3_	rt	48	57	>98:2
13	EtAlCl_2_	100	CHCl_3_	60	3	83	>98:2
14	EtAlCl_2_	100	toluene	60	5	94	>98:2

^a^Reaction conditions: A solution of **2** (0.22 mmol) and the catalyst in the appropriate solvent (2 mL, 0.1 M) was stirred at room temperature for 1 h, then **1a** (0.2 mmol) was added and the mixture stirred at the stated time and temperature. ^b^Starting materials recovered at the end of the reaction. ^c^Mixture of unidentified compounds.

The reaction was ineffective under thermal conditions (Table 1, entry 1). As a consequence we tested simple Lewis acids as potential promoters for the transformation. Magnesium perchlorate, scandium and copper triflate and boron trifluoride failed to give the desired compounds (Table 1, entries 2–7). Switching the reaction medium from toluene to chloroform (Table 1, entry 4), or increasing the catalyst loading, entry 7, were also ineffective. In all reactions tested the starting materials were recovered unreacted at the end of the reaction; decomposition was observed only using copper(II) triflate as catalyst (Table 1, entry 5). Unsatisfactory results were obtained also in the presence of gold(III) and gold(I) catalysts (Table 1, entries 8 and 9). Only in the presence of a cationic gold(I) complex the diastereoisomeric cycloadducts **3a** and **3'a** were isolated in 20% overall yield and in a diastereomeric ratio of 1:1 (Table 1, entry 9). These results were quite surprising as these catalyst/solvent systems were effective in our previously reported Diels–Alder cycloadditions involving **1a** as diene [18–21]. In particular, under gold catalysis excellent results in term of yields and selectivity were achieved [20–21]. We next turned our attention to aluminium catalysis. The use of 20 mol % of ethylaluminium dichloride in chloroform or toluene at room temperature resulted in the isolation of **3a** and **3'a** in 35% and 30% overall yield, respectively, in excellent diastereoisomeric ratios, higher than 98:2 in favour of the 2,3-*trans* adduct (Table 1, entries 10 and 11). Better yields could be obtained increasing the catalyst loading to 100 mol % (Table 1, entry 12) whereas the best results were achieved working under the same reaction conditions at 60 °C, in chloroform or toluene as solvents (Table 1, entries 13 and 14). Under these conditions **3a** and **3'a** were isolated in 83% and 94% overall yields, respectively, preserving the same diastereoisomeric ratios. With the best reaction conditions in hands, the scope of the transformation was then explored using the 2-vinylindoles **1a–j.** Results are shown in Table 2.

**Table 2:** Scope of the cycloaddition reactions between 2-vinylindoles **1a–j** and methyl 2-acetamidoacrylate (**2**).

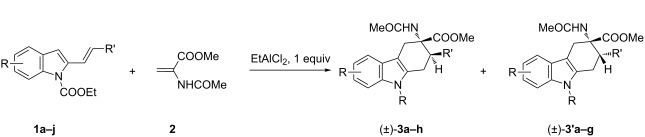							

Entry	2-Vinylindole	Solvent	*T*, °C	Time, h	Products	Yield, %	dr **3**:**3**'

1	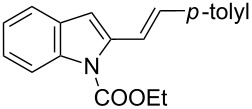 **1a**	CHCl_3_	60	3	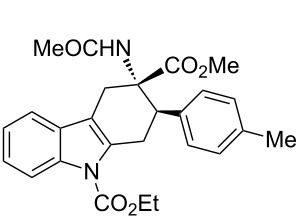 (±)-**3a**	83	>98:2
2	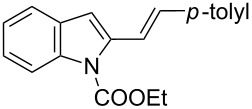 **1a**	toluene	60	5	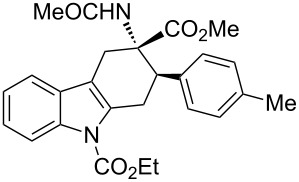 (±)-**3a**	94	>98:2
3	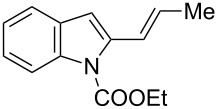 **1b**	CHCl_3_	60	3	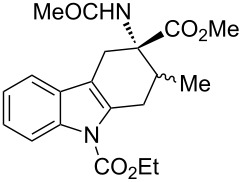 (±)-**3b**, (±)-**3'b**	86^a^	1:1
4	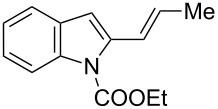 **1b**	toluene	60	3	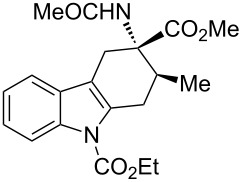 (±)-**3b**	84	>98:2
5	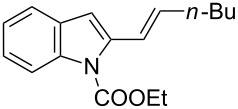 **1c**	toluene	60	4	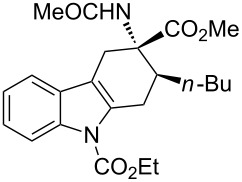 (±)-**3c**	74	>98:2
6	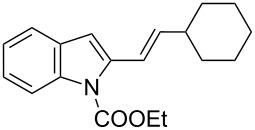 **1d**	toluene	60	4	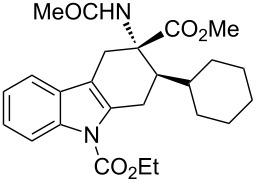 (±)-**3d**	83	>98:2
7	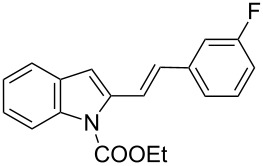 **1e**	toluene	60	6	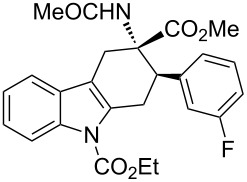 (±)-**3e**	79	>98:2
8	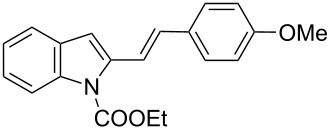 **1f**	toluene	60	5	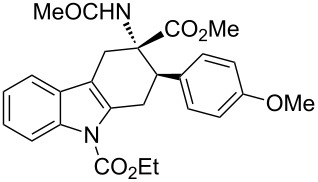 (±)-**3f**	78	>98:2
9	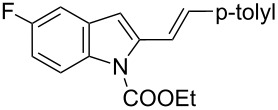 **1g**	toluene	60	24	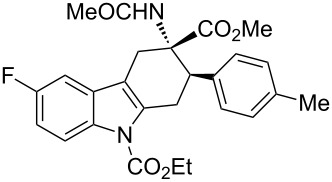 (±)-**3g**	50	>98:2
10	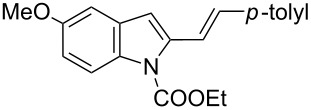 **1h**	toluene	60	24	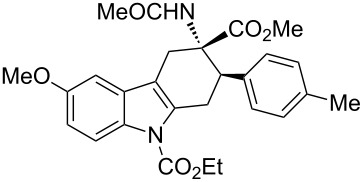 (±)-**3h**	46	>98:2
11	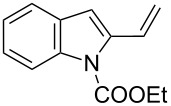 **1i**	toluene	60	5	–	–^b^	–
12	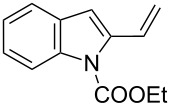 **1i**	toluene	rt	5	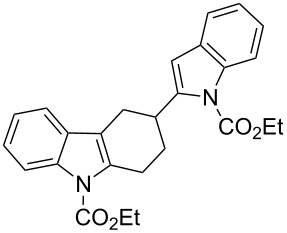 **4**	33	–
13	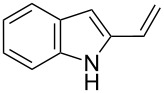 **1j**	toluene	rt	5	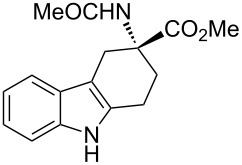 (±)-**3i**	44	–
14	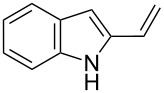 **1j**	CHCl_3_	rt	5	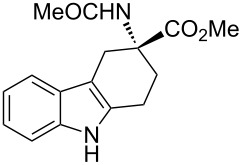 (±)-**3i**	12	–

^a^Overall yield (**3** + **3’**), pure isolated compounds after chromatographic purification. ^b^Mixture of unidentified compounds.

Table 2, entries 1 and 2 report the best results for the cycloaddition reaction of **1a** with **2**, obtained during the reaction conditions screening (see Table 1). Quite surprisingly, indole **1b** bearing a methyl group at the distal position of the diene system, gives rise to the desired cycloadducts **3b** and **3'b** in good yields but without any diastereoselectivity when we performed the reaction in chloroform (Table 2, entry 3). Nevertheless, switching from chloroform to toluene as reaction solvent, results in a total regain of diastereoselectivity without loss of efficiency (Table 2, entry 4). As a consequence, we choose toluene as solvent for further cycloaddition reactions. Under these conditions, alkyl- (Table 2, entries 5 and 6) and aryl- (entries 7 and 8) substituted dienes smoothly react with methyl 2-acetamidoacrylate (**2**) affording the desired cycloadducts in good yields and excellent diastereoselectivities. A considerable drop in yield is observed using dienes substituted in position 5 of the indole ring with an EWG or an EDG such as fluorine or methoxy (Table 2, entries 9 and 10). Finally, the indole **1i**, unsubstituted at the terminal position of the diene system, decomposes at 60 °C (Table 2, entry 11) whereas working at room temperature, entry 12, the main isolated compound, beside unreacted **1i** and **2**, is the vinylindole dimer **4** (33% yield) arising from the cycloaddition reaction between two molecules of vinylindole **1i**. However, the same reaction performed with the *N*-unsubstituted vinylindole **1j** in toluene or chloroform as solvents and at room temperature (Table 2, entries 13 and 14) allows for the isolation of the desired cycloadduct **3i**, in moderate and poor yields, respectively. A small quantity of a dimeric compound analogous to **4** was observed in the crude reaction mixture, via ^1^H NMR, along with a mixture of unidentified compounds.

**Figure 3:**
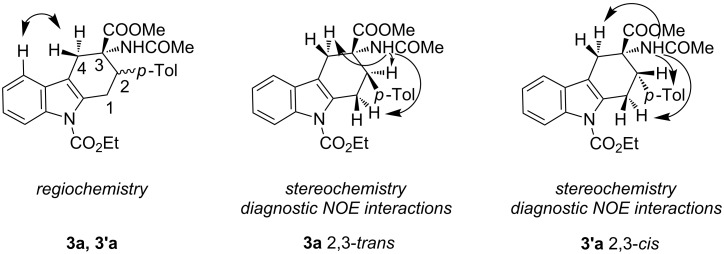
Structure elucidation of diastereoisomeric tetrahydrocarbazoles **3a** and **3’a** via NMR experiments.

The diastereoisomeric mixtures (**3**/**3'**) could be easily separated by flash chromatography and pure isomers were characterized by combined one- (^1^H NMR, ^13^C NMR-APT) and two-dimensional (COSY, HMBC, NOESY) experiments, performed at 300 MHz, using C_6_D_6_ or CDCl_3_ as solvents. In particular, the regiochemistry and stereochemistry around the C1/C4 moiety were assigned on the basis of spatial coupling interactions detected by 2D-NOESY experiments. As an example, the regiochemistry of the Diels–Alder adducts **3a** and **3'a** was reasonably assigned on the basis of NOE interactions between H-5 and the two H-4 hydrogens, Figure 3. Furthermore, for compound **3a** it was possible to observe diagnostic NOE interactions between NH and the three *cis* hydrogens in position 1, 2 and 4, Figure 3. On the contrary, for compound **3'a** in Figure 3, NH NOE interactions involve *cis* hydrogens at positions 1 and 4 and the aromatic *p*-tolyl hydrogens, Figure 3.

**Scheme 2:**
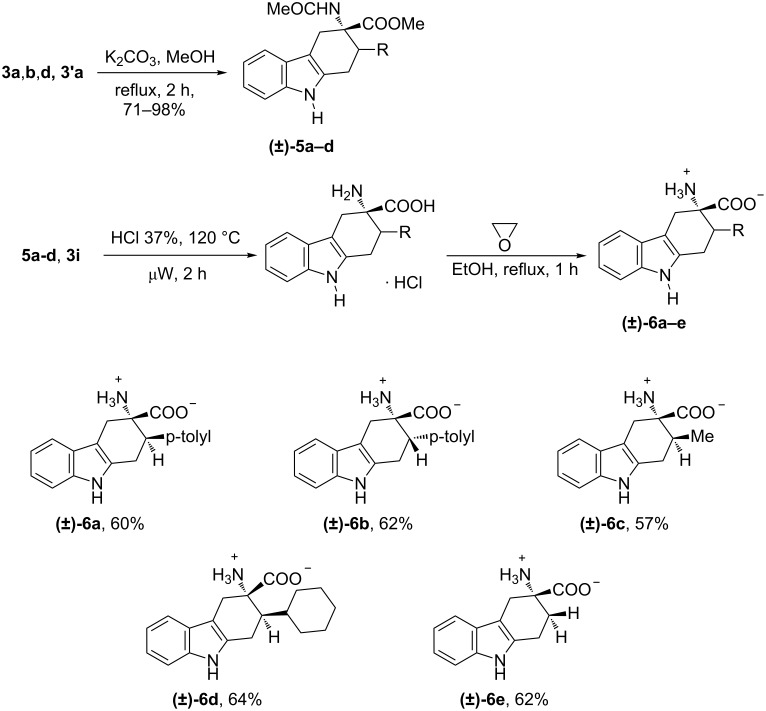
Synthesis of unprotected tryptophan derivatives **6a–e**.

Finally, tetrahydrocarbazoles **3a**,**b**,**d** and **3'a** were selected as substrates to test the reactivity of our compounds under hydrolytic conditions to obtain the deprotection of indole nitrogen and the free amino acid function at C-3, Scheme 2. The deprotection of indole nitrogen, giving rise to compounds **5a–d**, was achieved in high yields in the presence of 1 equiv of potassium carbonate, in methanol at reflux for 2 h. After characterisation, the obtained compounds **5a–d** and **3i** were treated first with hydrochloric acid (12 N) in a multimode microwave oven at 120 °C for 2 h, then with an excess of propylene oxide in ethanol at reflux for 1 h, and finally purified by flash chromatography. The whole reaction sequence was realized in a one-flask procedure. The obtained compounds **6a–e** and the relative reaction yields, referred to the starting compounds **3**/**3'**, are reported in Scheme 2.

No epimerisation reactions occurred during the hydrolytic processes as demonstrated via 2D NMR experiments (COSY, HSQC).

## Discussion

**Scheme 3:**
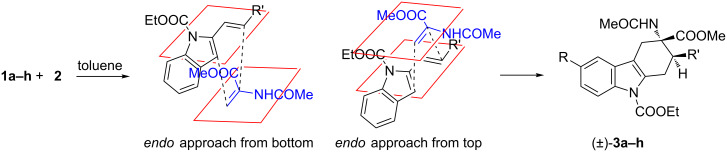
Plausible reaction mechanism for the cycloaddition reactions of indoles **1a–h** with **2** in toluene.

In the Diels–Alder reaction of vinylindoles **1** with methyl 2-acetamidoacrylate (**2**), among tested Lewis acid, only EtAlCl_2_ is able to trigger the reaction toward the formation of the desired cycloadducts **3**/**3'**. Similarly, Piersanti and co-workers reported the unique capability of EtAlCl_2_, with respect to related hard Lewis acids, to activate **2** toward the nucleophilic addition of indoles [29]. They ascribed the observed reactivity to the formation of a complex between EtAlCl_2_ and **2**, verified via ^1^H NMR experiments, and involving coordination with both amide and ester carbonyl groups [29]. Conceivably, the same activated complex participated in our Diels–Alder reactions, see footnote a in Table 1. Moreover, the high diastereoselectivity observed with indoles **1a–h** in toluene as solvent, accounts for a concerted or a pseudoconcerted mechanism in which the formation of an *endo* transition state and thus of the final 2,3-*trans* adducts **3a–h** is preferred (Scheme 3).

**Scheme 4:**
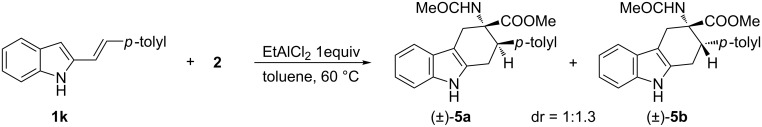
Cycloaddition reaction of 2-vinylindole **1k** and methyl 2-acetamidoacrylate (**2**).

Thus bond formation between the 1,4-carbon terminus of the diene and the double bond of the dienophile occurs almost simultaneously allowing for the preservation of the stereochemistry around the outer-ring diene double bond. Besides, in the presence of a terminal *p*-tolyl group on the diene moiety (see Table 2, entries 1 and 2) the diastereoselectivity is not affected by the reaction medium (chloroform vs toluene). Moving to the alkyl-substituted vinylindole **1b**, the reaction yields equimolecular amounts of both conceivable diastereoisomers in CHCl_3_ whereas toluene is the optimal solvent to achieve high diastereoselection (see Table 2, entries 3 and 4). It is well known that reaction rates and selectivity in Diels–Alder reactions are affected by solvents [30–32]. In particular, polarity and hydrogen bond donor ability of the solvent can impact the diastereoisomeric ratios in a Diels–Alder reaction. Such an effect seems negligible in the reaction of **1a** with **2** and effective in the reaction of **1b** with **2**. In this latter case, the reaction outcome could be explained by the formation of two energetically comparable transition states (*endo* + *exo* TS) or by the occurrence of a stepwise mechanism. Finally, the results obtained with indole **1i**, unsubstituted at the terminal position of the diene system, can be attributed to the thermal instability of **1i** under standard reaction conditions and to the lack of reactivity toward **2** at room temperature. The lack of reactivity of **1i** toward **2** can be overcome working with *N*-unprotected indole **1j**. Modulations in reactivity upon specific substitution patterns at the indole nucleus were observed by us in competitive Diels–Alder cycloaddition/Michael addition reactions of vinylindoles with classical dienophile and in competitive cycloaddition/hydroarylation reactions with allenes, see [20–21] for a more detailed discussion. Conceivably, removal of the EWG at the nitrogen modifies the electronic distribution of the reacting diene allowing the desired reaction to take place. Moreover, a model reaction performed with the 2-(4-methylstyryl)-1*H*-indole (**1k**), unprotected at N1 and comparable to **1a** at the diene moiety, and **2** afforded the corresponding tetrahydrocarbazoles **5a** and **5b** in 1:1.3 diasteroisomeric ratio (Scheme 4).

As reported for the reaction between **1b** and **2** in chloroform, the reaction outcome could be explained by the formation of two energetically comparable transition states (*endo* + *exo* TS) or by the occurrence of a stepwise mechanism. Distinction between the two proposed mechanisms cannot be made on the basis of experimental evidences. Therefore, isolation or detection in the reaction mixtures of plausible intermediates were unsuccessful and a computational study devoted to the identification of the most suitable transition state is beyond the scope of this work.

## Conclusion

In summary, we reported a flexible approach to the synthesis of an important class of compounds like constrained tryptophan derivatives, pertinent to the class of tetrahydrocabazoles. The synthesis was realised through an intermolecular [4 + 2]-cycloaddition of 2-vinylindoles and methyl 2-acetamidoacrylate. Although the reaction requires a stoichiometric amount of EtAlCl_2_ as promoter, it presents several advantages with respect to classical Fischer indole synthesis, normally adopted for the preparation of these derivatives [11–13]. First of all, modulation of substituents around the terahydrocarbazole nucleus is achievable without the formation of regioisomeric derivatives. Moreover, there is no need of multistep sequences for the synthesis of starting materials. Methyl 2-acetamidoacrylate is a commercially available and cheap reactant, whereas 2-vinylindoles can be easily synthesized by a common precursor [22]. Finally, by exploring the scope of the reaction and in connection with our previous reports on the cycloaddition reactions of 2-vinylindoles, we were able to point out several features about the reactivity of these compounds. In particular, the dependence upon the substitution pattern at nitrogen and at the outer-ring double bond, highlighted the need to select the appropriate promoter for each desired transformation.

## Supporting Information

File 1 Experimental procedures and analytical data.
