# Supplementary material for: Synthesis of constrained analogues of tryptophan
Source: Beilstein J Org Chem. 2015 Oct 27;11:1997–2006. doi: 10.3762/bjoc.11.216 (PMC4661003; doi:10.3762/bjoc.11.216)
Supplement: File 1 — Experimental procedures and analytical data. [file Beilstein_J_Org_Chem-11-1997-s001.pdf]

Supporting Information

for

Synthesis of constrained analogues of tryptophan

Elisabetta Rossi*, Valentina Pirovano*, Marco Negrato, Giorgio Abbiati and Monica Dell'Acqua

Address: Dipartimento di Scienze Farmaceutiche, Sezione di Chimica Generale e Organica "A. Marchesini", Università degli Studi di Milano, Via Venezian, 21, 20133 Milano, Italy

Email: Elisabetta Rossi - elisabetta.rossi@unimi.it

* Corresponding authors

Experimental procedures and analytical data

Contents

General remarks s2

Preparation and characterization data for compounds **(±)-3a–h**, **(±)-3'a,b** and **(±)-4** s3

Preparation and characterization data for compounds **(±)-3i, (±)-5a, (±)-5b** s9

Preparation and characterization data for compounds **(±)-5a–d** s10

Preparation and characterization data for compounds **(±)-6a,e** s12

2D NMR experiments s15

References s23

General Remarks: All chemicals and solvents are commercially available and were used after distillation or treatment with drying agents. Silica gel F254 thin-layer plates were employed for thin-layer chromatography (TLC). Silica gel 40–63 micron/60 Å was employed for flash column chromatography. Melting points were measured with a Perkin-Elmer DSC 6 calorimeter at a heating rate of 5 °C/min and are uncorrected. ^{1}H and ^{13}C NMR spectra were determined with a Varian-Gemini 200, a Bruker 300 or 500 Avance spectrometers at room temperature in $CDCl_3$, DMSO-d_6 or D_2O with residual solvent peaks as the internal reference. The APT sequences were used to distinguish the methine and methyl carbon signals from those arising from methylene and quaternary carbon atoms. Two-dimensional NMR experiments were performed, where appropriate, to aid the assignment of structures. Low-resolution MS spectra were recorded with a Thermo-Finnigan LCQ advantage AP electrospray/ion trap equipped instrument using a syringe pump device to directly inject sample solutions.

This study was carried out using (*E*)-2-vinylindoles **1a–g, 1j**,[1] **1h**[2] and methyl 2-acetamidoacrylate (**2**),[3] which are known compounds and were prepared according to standard procedures.

$Mg(ClO_4)_2$, $Sc(OTf)_3$, $Cu(OTf)_2$, $BF_3{\cdot}OEt_2$, $AuCl_3$, $[Au(PPh_3)Cl]$ and $EtAlCl_2$ were purchased from commercial suppliers and used as received.

Preparation and characterization data for compounds (±)-3a–h, (±)-3’a,b and (±)-4

A N_2-flushed solution of ethylaluminium dichloride (1.0 M in hexane, 1.0 equiv) and methyl 2-acetamidoacrylate (**2**) (1.10 equiv) in anhydrous toluene (0.1 M) was stirred at room temperature for 1 h. After this time, (*E*)-2-vinylindole **1a–h** (1.00 equiv) was added and the mixture was heated at 60 °C for the required time. Then, the mixture was cooled to room temperature and quenched with Na_2HCO_3 sat. sol. The aqueous layer was extracted with ethyl acetate (3 ×). The combined organic layers were dried over Na_2SO_4, filtered, and the solvent evaporated under vacuum. The crude was purified by flash chromatography (SiO_2, hexane/ethyl acetate 2:1).

(±)-*trans*-9-Ethyl 3-methyl 3-acetamido-2-(*p*-tolyl)-3,4-dihydro-1*H*-carbazole-3,9(2*H*)-dicarboxylate (3a)

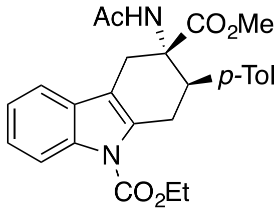

General procedure was followed using **1a** (61.1 mg, 0.2 mmol), **2** (31.5 mg, 0.22 mmol), $EtAlCl_2$ (0.2 mL, 0.2 mmol) in toluene (2 mL). Product **3a** (85.2 mg, 94%) was obtained as main reaction product as a white solid (m.p. 191.2–195.6 °C).
^{1}H NMR (300 MHz, C_6D_6): δ = 8.47 (d, *J* = 8.4 Hz, 1H), 7.34 (m, 2H), 7.27 (m, 3H), 7.00 (d, *J* = 7.9 Hz, 2H), 6.12 (bs, 1H), 4.28 (d, *J* = 15.1 Hz, 1H), 4.08 (m, 3H), 3.78 (dd, *J* = 5.8, 14.5 Hz, 1H), 3.63 (dd, *J* = 6.5, 14.5 Hz, 1H), 3.53 (d, *J* = 15.1 Hz, 1H), 2.41 (s, 3H), 2.16 (s, 3H), 1.02 (t, *J* = 7.0 Hz, 3H) ppm. **^{13}C NMR APT** (75 MHz, $CDCl_3$): δ =172.8 (C), 170.0 (C), 152.2 (C), 137.6 (C), 136.7 (C), 136.4 (C), 132.5 (C), 129.7 (C), 129.5 (2xCH), 128.1 (2xCH), 124.4 (CH), 123.2 (CH), 118.4 (CH), 115.8 (CH), 63.2 (CH_2), 62.1 (C), 52.6 (CH_3), 45.0 (CH), 29.5 (CH_2), 25.5 (CH_2), 23.9 (CH_3), 21.2 (CH_3), 14.6 (CH_3) ppm. **ESI(+)-MS**: m/z (%) = 435 (100) $[M + H]^+$; $C_{26}H_{28}N_2O_5$ [448.52]: calcd. for C 69.63, H 6.29, N 6.25; found C 69.93, H 6.44, N 6.45.

(±)-*cis*-9-Ethyl 3-methyl 3-acetamido-2-(*p*-tolyl)-3,4-dihydro-1*H*-carbazole-3,9(2*H*)-dicarboxylate (3’a)

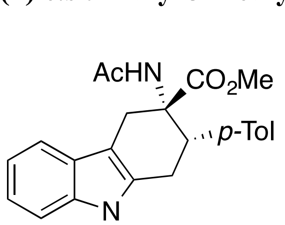

Product **3’a** was isolated in traces following the reported general procedure. In alternative it could be isolated using $Au(PPh_3)Cl/AgOTf$ (2 mol %) as catalyst (see Table 1).

^{1}H NMR (300 MHz, $CDCl_3$): δ = 8.19 (dd, *J* = 1.0, 7.2 Hz, 1H), 7.45 (m, 1H), 7.31 (m, 2H), 7.12 (m, 4H), 5.57 (bs, 1H), 4.48 (q, *J* = 7.0 Hz, 2H), 3.85 (dd, *J* = 4.5, 6.5 Hz, 1H), 3.75-3.66 (m, 2H), 3.61 (s, 3H), 3.44 (dd, *J* = 4.5, 18.5 Hz, 1H), 3.02 (d, *J* = 16.1 Hz, 1H), 2.35 (s, 3H), 1.91 (s, 3H), 1.47 (t, *J* = 7.0 Hz, 3H) ppm. **^{13}C NMR APT** (75 MHz, $CDCl_3$): δ =172.7 (C), 170.1 (C), 152.1 (C), 137.7 (C), 137.6 (C), 136.5 (C), 133.2 (C), 129.8 (2xCH), 129.2 (C), 128.4 (2xCH), 124.3 (CH), 123.1 (CH), 118.2 (CH), 115.8 (CH), 114.5 (C), 63.2 (CH_2), 60.9 (C), 52.7 (CH_3), 44.4 (CH), 29.9 (CH_2), 27.9 (CH_2), 23.5 (CH_3), 21.2 (CH_3), 14.6 (CH_3) ppm.

(±)-*trans*-9-Ethyl 3-methyl 3-acetamido-2-methyl-3,4-dihydro-1*H*-carbazole-3,9(2*H*)-dicarboxylate (3b)

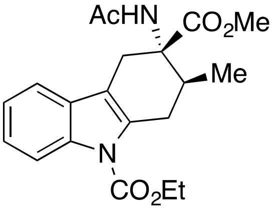

General procedure was followed using **1b** (57.3 mg, 0.25 mmol), **2** (39.4 mg, 0.27 mmol) and $EtAlCl_2$ (0.25 mL, 0.25 mmol) in toluene (2.5 mL). Product **3b** (78.2 mg, 84%) was obtained as white solid (m.p. 173.0-176.8 °C).

^{1}H NMR (300 MHz, $CDCl_3$): δ = 8.10 (d, *J* = 7.7 Hz, 1H), 7.45 (d, *J* = 7.0 Hz, 1H), 7.33-7.20 (m, 2H), 5.96 (bs, 1H), 4.49 (q, *J* = 7.3 Hz, 2H), 3.78 (s, 3H), 3.36-3.22 (m, 3H), 3.06 (d, *J* = 19.0 Hz, 1H), 2.61 (t, *J* = 7.2 Hz, 1H), 1.91 (s, 3H), 1.50 (t, *J* = 7.0, 3H), 1.01 (d, *J* = 7.0, 3H) ppm. **^{13}C NMR APT** (75 MHz, $CDCl_3$): δ = 173.4 (C), 170.1 (C), 152.4 (C), 136.4 (C), 131.4 (C), 129.9 (C), 124.4 (CH), 123.3 (CH), 118.5 (CH), 115.9 (CH), 114.5 (C), 63.4 (CH_2), 61.28 (C), 52.9 (CH_3), 34.2 (CH), 30.1 (CH_2), 24.8 (CH_2), 23.8 (CH_3), 16.5 (CH_3), 14.8 (CH_3). **ESI(+)-MS**: m/z (%) = 373 (100) $[M + H]^+$; $C_{20}H_{24}N_2O_5$ [372.41]: calcd. for C 64.50, H 6.50, N 7.52; found C 64.62, H 6.44, N 7.30.

(±)-*cis*-9-Ethyl 3-methyl 3-acetamido-2-methyl-3,4-dihydro-1*H*-carbazole-3,9(2*H*)-dicarboxylate (3'b)

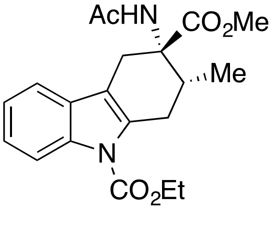

Product **3'b** was isolated in 43% yield using $CHCl_3$ as solvent (see Table 2).

^{1}H NMR (200 MHz, DMSO-*d_6*): δ = 8.45 (s, 1H), 8.06 (d, *J* = 7.7 Hz, 1H), 7.76-7.23 (m, 2H), 4.44 (q, *J* = 7.2 Hz, 2H), 3.57 (s, 3H), 3.17 (dd, *J* = 4.2, 18.3 Hz, 1H), 2.47 (m, 2H), 1.98 (m, 2H), 1.81 (s, 3H), 1.38 (t, *J* = 7 Hz, 3H), 1.06 (d, *J* = 6.1 Hz, 3H) ppm. **^{13}C NMR APT** (DMSO-*d_6*, 50 MHz): δ = 172.2 (C), 169.3 (C), 151.7 (C), 139.8 (C), 136.1 (C), 127.5 (C), 124.4 (CH), 123.4 (CH), 120.8 (CH), 115.5 (CH), 114.8 (C), 64.0

(CH_2), 59.1 (C), 52.7 (CH_3), 34.5 (CH_2), 26.0 (CH), 23.3 (CH_3), 21.9 (CH_3), 14.7 (CH_3) ppm. 1 CH_2 is overlapping.

(±)-*trans*-9-Ethyl 3-methyl 3-acetamido-2-butyl-3,4-dihydro-1*H*-carbazole-3,9(2*H*)-dicarboxylate (3c)

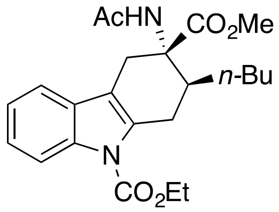

General procedure B was followed using **1c** (109 mg, 0.4 mmol), **2** (63.0 mg, 0.44 mmol) and $EtAlCl_2$ (0.4 mL, 0.4 mmol) in toluene (4.0 mL). Product **3c** (116 mg, 74%) was obtained as white solid (m.p. 154.9-158.3 °C).

^{1}**H NMR** (200 MHz, $CDCl_3$): δ = 8,08 (dd, *J* = 7.4 Hz, 1.2 1H), 7.44-7.20 (m, 3H), 6.03 (bs, 1H), 4.49 (q, *J* = 7.3 Hz, 2H), 3.78 (s, 3H), 3.40 (d, *J* = 16.7, 1H), 3.29-3.16 (m, 3H), 2.39 (m, 1H), 1.90 (s, 3H), 1.49 (t, *J* = 7.0, 3H), 1.53-1.12 (m, 6H), 0.87 (d, *J* = 6.6, 3H) ppm. ^{13}**C NMR APT** (50 MHz, $CDCl_3$): δ = 173.4 (C), 169.8 (C), 152.3 (C), 136.2 (C), 131.7 (C), 129.8 (C), 124.3 (CH), 123.2 (CH), 118.3 (CH), 115.7 (CH), 114.7 (C), 63.2 (CH2), 61.3 (C), 52.7 (CH_3), 39.3 (CH), 30.2(CH_2), 29.0 (CH_2), 26.5 (CH_2), 25.1 (CH_2), 23.6 (CH_3), 22.7 (5CH_2), 14.6 (CH_3), 14.1 (CH_3) ppm. **ESI(+)-MS**: m/z (%) = 415 (21) $[M + H]^+$, 437 (21) $[M + Na]^+$; $C_{23}H_{30}N_2O_5$ [414.50]: calcd. for C 66.65, H 7.30, N 6.76; found C 66.78, H 7.42, N 6.82.

(±)-*trans*-9-Ethyl 3-methyl 3-acetamido-2-cyclohexyl-3,4-dihydro-1*H*-carbazole-3,9(2*H*)-dicarboxylate (3d)

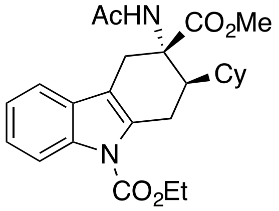

General procedure was followed using **1d** (100 mg, 0.34 mmol), **2** (53.0 mg, 0.37 mmol) and $EtAlCl_2$ (0.34 mL, 0.34 mmol) in toluene (3.4 mL). Product **3d** (124 mg, 83%), was obtained as white solid (m.p. 199.3–205.2 °C).

^{1}**H NMR** (300 MHz, $CDCl_3$): δ = 8.07 (d, *J* = 7.4 Hz, 1H), 7.41-7.18 (m, 3H), 6.21 (bs, 1H), 4.50 (q, *J* = 6.96 Hz, 2H), 3.76 (s, 3H), 3.56 (d, *J* = 17.7, 1H), 3.31 (dd, *J* = 4.4, 19.3 Hz, 1H), 3.25-3.10 (m, 2H), 2.43 (t, *J* = 4.9 Hz, 1H), 1.93 (s, 3H), 1.75 (m, 1H), 1.66-0.84 (m, 13H) ppm. ^{13}**C NMR APT** (75 MHz, $CDCl_3$): δ = 174.1 (C), 169.7 (C), 152.5 (C), 136.2 (C), 133.5 (C), 129.9 (C), 124.3 (CH), 123.2 (CH), 118.4 (CH), 115.9 (CH), 115.3 (C), 63.3 (CH_2), 61.8 (C), 53.0 (CH_3), 44.7 (CH), 38.1 (CH), 34.6 (CH_2), 28.5 (CH_2), 27.5 (CH_2), 26.9 (CH_2), 26.8 (CH_2), 26.5 (CH_2), 24.1 (CH_3), 23.8 (CH_2), 14.8 (CH_3) ppm. **ESI(+)-MS**: m/z (%) = 441 (100) $[M + H]^+$. $C_{25}H_{32}N_2O_5$ [440.54]: calcd. for C 68.16, H 7.32, N 6.36; found C.68.45, H 7.21, N 6.48.

(±)-*trans*-9-Ethyl 3-methyl 3-acetamido-2-(3-fluorophenyl)-3,4-dihydro-1*H*-carbazole-3,9(2*H*)-dicarboxylate (3e)

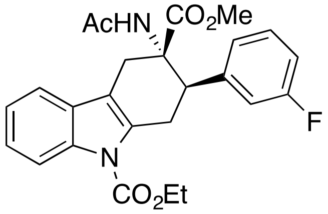

General procedure was followed using **1e** (61.9 mg, 0.2 mmol), **2** (31.5 mg, 0.22 mmol) and $EtAlCl_2$ (0.2 mL, 0.2 mmol) in toluene (2 mL). Product **3e** (78.6 mg, 79%) was obtained as a white solid (m.p. 193.0-197.5 °C).

^{1}H NMR (200 MHz, $CDCl_3$): δ = 8.14 (dd, *J* = 6.7, 1.2 Hz, 1H), 7.45-7.15 (m, 4H), 6.98-6.77 (m, 3H), 6.13 (bs, 1H), 4.49 (q, *J* = 6.8 Hz, 2H), 3.87 (t, *J* = 6.2 Hz, 1H), 3.73-3.45 (m, 6H), 3.13 (d, *J* = 17.2 Hz, 1H), 1.93 (s, 3H), 1.48 (t, *J* = 7.0 Hz, 3H) ppm. **^{13}C NMR APT** (50 MHz, $CDCl_3$): δ = 172.6 (C), 170.1 (C), 162.9 (d, $^1J_{C\text{-}F}$ = 240 Hz, C), 152.2 (C), 142.4 (d, $^3J_{C\text{-}F}$ = 7.2 Hz, C), 136.3 (C), 132.2 (C), 130.2 (d, $^3J_{C\text{-}F}$ = 8.4 Hz, CH), 129.6 (C), 124.5 (CH), 124.1 (d, $^4J_{C\text{-}F}$ = 3.0 Hz, CH), 123.3 (CH), 118.4 (CH), 115.9 (CH), 115.6 (C), 115.3 (d, $^2J_{C\text{-}F}$ = 14.8 Hz, CH), 114.8 (d, $^2J_{C\text{-}F}$ = 13.7 Hz, CH), 63.3 (CH_2), 62.0 (C), 52.7 (CH_3), 44.9 (CH), 29.4 (CH_2), 25.4 (CH_2), 23.9 (CH_3), 14.5 (CH_3) ppm. **ESI(+)-MS**: m/z (%) = 453 (36) $[M + H]^+$; 475 (100) $[M + Na]^+$. $C_{25}H_{25}FN_2O_5$ [452.48]: calcd. for C 66.36, H 5.57, N 6.19; found: C 66.48, H 5.61, N 6.02.

(±)-*trans*-9-Ethyl 3-methyl 3-acetamido-2-(4-methoxyphenyl)-3,4-dihydro-1*H*-carbazole-3,9(2*H*)-dicarboxylate (3f)

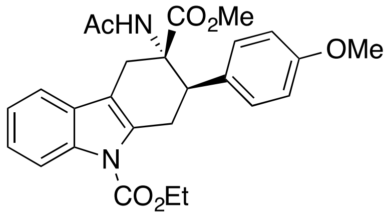

General procedure was followed using **1f** (64.3 mg, 0.2 mmol), **2** (31.5 mg, 0.22 mmol) and $EtAlCl_2$ (0.2 mL, 0.2 mmol) in toluene (2 mL). Product **3f** (72.5 mg, 78%) was obtained as white wax.

^{1}H NMR (200 MHz, $CDCl_3$): δ = 8.16 (dd, *J* = 7.0, 1.1 Hz, 1H), 7.45 (m, 1H), 7.36-7.26 (m, 2H), 6.99 (d, *J* = 8.8 Hz, 2H), 6.75 (d, *J* = 8.8 Hz, 2H), 6.09 (bs, 1H), 4.46 (q, *J* = 7.0 Hz, 2H), 3.75 (s, 3H), 3.71-3.50 (m, 7H), 3.16 (d, *J* = 17.2 Hz, 1H), 1.92 (s, 3H), 1.45 (t, *J* = 7.0 Hz, 3H) ppm. **^{13}C NMR APT** (50 MHz, $CDCl_3$): δ = 172.8 (C), 170.1 (C), 159.2 (C), 152.2 (C), 136.4 (C), 132.5 (C), 131.8 (C), 129.7 (C), 129.3 (2xCH), 124.4 (CH), 123.2 (CH), 118.4 (CH), 115.8 (CH), 115.7 (C), 114.1 (2xCH), 63.3 (CH_2), 62.1 (C), 55.3 (CH_3), 52.6 (CH_3), 44.6 (CH), 29.6 (CH_2), 25.2 (CH_2), 23.8 (CH_3), 14.6 (CH_3) ppm. **ESI(+)-MS**: m/z (%) =

463 (20) [M - H]$^+$, 405 (100) [M - COOEt]$^+$. $C_{26}H_{28}N_2O_6$ [464.52]: calcd. for C 67.23, H 6.08, N 6.03; found C 67.48, H 6.12, N 5.83.

(±)-*trans*-9-Ethyl 3-methyl 3-acetamido-6-fluoro-2-(*p*-tolyl)-3,4-dihydro-1*H*-carbazole-3,9(2*H*)-dicarboxylate (3g)

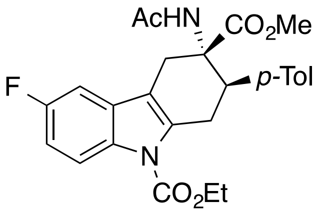

General procedure was followed using **1g** (64.3 mg, 0.2 mmol), **2** (31.5 mg, 0.22 mmol) and $EtAlCl_2$ (0.2 mL, 0.2 mmol) in toluene (2 mL). Product **3g** (46.7 mg, 50%) was obtained as white solid (m.p. 225.1–228.5 °C).

^{1}H NMR (200 MHz, $CDCl_3$): δ = 8.10 (dd, *J* = 4.8, 9.2 Hz, 1H), 7.10-7.01 (m, 4H), 6.94 (d, *J* = 8.4 Hz, 2H), 6.09 (bs, 1H), 4.47 (q, *J* = 7.0 Hz, 2H), 3.82 (t, *J* = 6.2, 1H), 3.66-3.52 (m, 6H), 3.2 (d, *J* = 17.0 Hz, 1H), 2.30 (s, 3H), 1.93 (s, 3H), 1.46 (t, *J* = 7.0 Hz, 3H) ppm. **^{13}C NMR APT** (50 MHz, $CDCl_3$): δ = 172.6 (C), 170.0 (C), 159.7 (d, $^1J_{C-F}$ = 240 Hz, C), 151.9 (C), 137.7 (C), 136.5 (C), 134.4 (C), 132.6 (C), 130.7 (d, $^3J_{C-F}$ = 9.5 Hz, C), 129.5 (2xCH), 128.1 (2xCH), 116.7 (d, $^3J_{C-F}$ = 8.7 Hz, CH), 115.5 (d, $^4J_{C-F}$ = 3.8 Hz, C), 111.7 (d, $^2J_{C-F}$ = 24.7 Hz, CH), 104.2 (d, $^2J_{C-F}$ = 24.0 Hz, CH), 63.4 (CH_2), 62.1 (C), 52.6 (CH_3), 44.77 (CH), 29.7 (CH_2), 25.8 (CH_2), 24.0 (CH_3), 21.3 (CH_3), 14.6 (CH_3) ppm. **ESI(+)-MS**: m/z (%) = 489 (100) [M + Na]$^+$. $C_{26}H_{27}FN_2O_5$ [466.51]: calcd. for C 66.94, H 65.83, N 6.00; found C 67.28, H 65.67, N 6.22.

(±)-*trans*-9-Ethyl 3-methyl 3-acetamido-6-methoxy-2-(*p*-tolyl)-3,4-dihydro-1*H*-carbazole-3,9(2*H*)-dicarboxylate (3h)

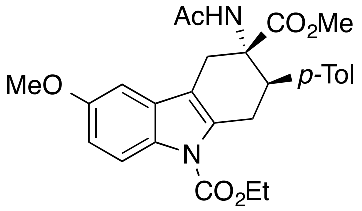

General procedure was followed using **1h** (67.1 mg, 0.2 mmol), **2** (31.5 mg, 0.22 mmol) and $EtAlCl_2$ (0.2 mL, 0.2 mmol) in toluene (2 mL). Product **3h** (43.8 mg, 46%) was obtained as white solid (m.p. 251.5-255 °C, dec.).

^{1}H NMR (200 MHz, DMSO-*d*$_6$): δ = 8.14 (bs, 1H), 7.93 (d, *J* = 9.2 Hz, 1H), 6.80-7.15 (m, 6H), 4.39 (q, *J* = 7.0 Hz, 2H), 3.77 (m, 4H), 3.66 (d, *J* = 14.3 Hz, 2H), 3.44 (s, 3H), 3.16 (d, *J* = 19.4 Hz, 1H), 2.82 (d, *J* = 17.6 Hz, 1H), 2.19 (s, 3H), 1.77 (s, 3H), 1.35 (t, *J* = 7.0 Hz, 3H) ppm. **^{13}C NMR APT** (50 MHz, DMSO-*d*$_6$): δ = 172.6 (C), 170.4 (C), 156.5 (C), 151.7 (C), 138.2 (C), 137.0 (C), 134.6 (C), 130.3 (C), 129.9 (2xCH),

128.1 (2xCH), 116.7 (CH), 114.5 (C), 112.7 (CH), 102.0 (CH), 63.6 (CH_2), 62.6 (C), 56.1 (CH_3), 52.3 (CH_3), 43.8 (CH), 27.8 (CH_2), 24.6 (CH_2), 23.2 (CH_3), 21.3 (CH_3), 14.8 (CH_3) ppm. **ESI(+)-MS**: m/z (%) = 479 (100) $[M + H]^+$, 501 (80) $[M + Na]^+$. $C_{27}H_{30}N_2O_6$ [478.54]: calcd. for C 67.77, H 6.32, N 5.85; found C 67.85, H 6.15 N 5.99.

(±)-Ethyl 3-(1-(ethoxycarbonyl)-1*H*-indol-2-yl)-1,2,3,4-tetrahydro-9*H*-carbazole-9-carboxylate (4)

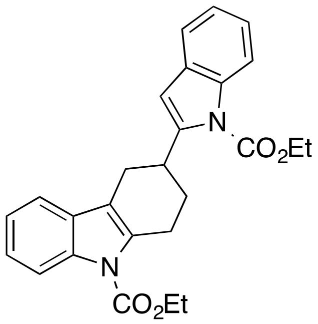

General procedure was followed using **1i** (43.05 mg, 0.2 mmol), **2** (31.5 mg, 0.22 mmol) and $EtAlCl_2$ (0.2 mL, 0.2 mmol) in toluene (2 mL) at room temperature. Product **4** (28.4 mg, 33%) was obtained as yellow wax.

^{1}H NMR (300 MHz, $CDCl_3$): δ = 8.15 (m, 2H), 7.01-7.32 (m, 6H), 5.97 (s, 1H), 5.12 (s, 1H), 4.42-4.46 (m, 4H), 3.24 (m, 1H), 2.94 (m, 1H), 2.07 (m, 2H), 1.82 (m, 2H), 1.40-1.58 (m, 6H). **^{13}C NMR APT** (75 MHz, $CDCl_3$): δ = 152.5 (C), 143.8 (C), 137.9 (C), 137.4 (C), 136.4 (C), 129.7 (C), 129.6 (C), 124.1 (CH), 124.0 (CH), 123.3 (CH), 123.1 (CH), 120.6 (CH), 119.1 (CH), 118.2 (C), 116.2 (CH), 115.9 (CH), 111.3 (CH), 63.6 (CH_2), 63.3 (CH_2), 32.3 (CH), 29.2 (CH_2), 26.2 (CH_2), 19.2 (CH_2), 14.9 (CH_3), 14.8 (CH_3) ppm. **ESI(+)-MS**: m/z (%) = 431 (100) $[M + H]^+$. $C_{26}H_{26}N_2O_4$ [430.50]: calcd. for C 72.54, H 6.09, N 6.51; found C 72.86, H 5.80, N 6.23.

Preparation and characterization data for compounds (±)-3i, (±)-5a, (±)-5b

(±)-Methyl 3-acetamido-2,3,4,9-tetrahydro-1*H*-carbazole-3-carboxylate (3i)

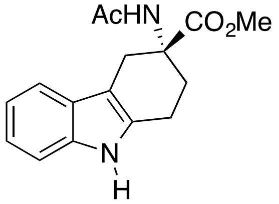

A N_2-flushed solution of ethylaluminium dichloride (0.70 mL, 0.70 mmol) and methyl 2-acetamidoacrylate (**2**) (110 mg, 0.77 mmol) in anhydrous toluene (7 mL) was stirred at room temperature for 1 h. After this time **1j** (100 mg, 0.70 mmol) was added and the mixture was left to stir at room temperature for 5 h.
Then, the mixture was cooled to room temperature and quenched with Na_2HCO_3 sat. sol. The aqueous layer was extracted with ethyl acetate (3 × 5 mL). The combined organic layers were dried over Na_2SO_4, filtered, and the solvent evaporated under vacuum. The crude was purified by flash chromatography (SiO_2, hexane/ethyl acetate 1:1) to yield **3i** (48.1 mg, 44%) as a white solid (m.p. 68.5–73.0 °C).
^{1}H NMR (200 MHz, DMSO-d_6): δ = 10.72 (bs, 1H), 8.12 (bs, 1H), 7.33 (d, J = 7.6 Hz, 1H), 7.25 (d, J = 7.3 Hz, 1H) 7.03-6.88 (m, 2H), 3.60 (s, 3H), 2.94 (s, 2H), 2.65 (m, 2H), 2.03 (m, 2H), 1.76 (s, 3H) ppm. **^{13}C NMR** (75 MHz, DMSO-d_6): δ = 175.1 (C), 170.3 (C), 136.8 (C), 133.6 (C), 128.0 (C), 121.0 (CH), 118.8 (CH), 117.9 (CH), 111.3 (CH), 105.3 (C), 57.9 (C), 52.6 (CH_3), 30.8 (CH_2), 28.6 (CH_2), 22.9 (CH_3), 19.7 (CH_2) ppm. **ESI(+)-MS**: m/z (%) = 287 (100) $[M + H]^+$. $C_{16}H_{18}N_2O_3$ [286.33], 309 (43) $[M + Na]^+$: calcd. for C 67.12, H 6.34, N 9.78; found C.67.34, H 6.21, N 9.83.

(±)-Methyl 3-acetamido-2-(*p*-tolyl)-2,3,4,9-tetrahydro-1*H*-carbazole-3-carboxylate (5a, 5b)

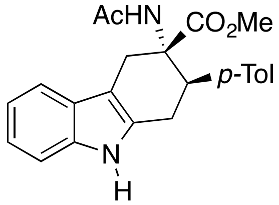

and

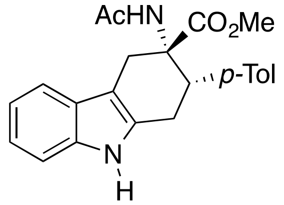

A N_2-flushed solution of ethylaluminium dichloride (0.43 mL, 0.43 mmol) and methyl 2-acetamidoacrylate (**2**) (61.0 mg, 0.43 mmol) in anhydrous $CHCl_3$ (4.3 mL) was stirred at room temperature for 1 h. After this time, **1k** (100 mg, 0.43 mmol) was added and the mixture was left to stir at 60 °C for 2 h.
Then, the mixture was cooled to room temperature and quenched with Na_2HCO_3 sat. sol. The aqueous layer was extracted with ethyl acetate (3 × 5 mL). The combined organic layers were dried over Na_2SO_4, filtered, and the solvent evaporated under vacuum. The crude was purified by flash chromatography (SiO_2, hexane/ethyl acetate 1:1) to yield respectively **5b** (61.0 mg, 37%) and **5a** (47.0 mg, 29%).
Characterisation of **5b** and **5a** is reported in the next section.

Preparation and characterization data for compounds (±)-5a–d

To a N_2-flushed stirring solution of K_2CO_3 (1.05 equiv) in methanol (0.05 M), the corresponding ethyl 3,4-dihydro-1*H*-carbazole-9(2*H*)-carboxylate **3a**, **3’a**, **3b**, **3d** (1.00 equiv) was added and the mixture was heated at 65 °C for 2 h. After that time the mixture was cooled to room temperature and solvent was evaporated. The residue was dissolved in ethyl acetate and water and the aqueous phase was extracted with ethyl acetate (3 ×). The combined organic layers were dried over Na_2SO_4, filtered, and the solvent evaporated under vacuum.

(±)-*trans*-Methyl 3-acetamido-2-(p-tolyl)-2,3,4,9-tetrahydro-1*H*-carbazole-3-carboxylate (5a)

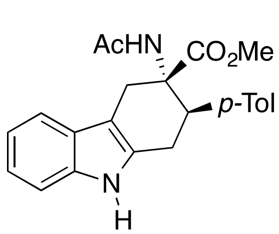

General procedure was followed using **3a** (200 mg, 0.45 mmol) and K_2CO_3 (65 mg, 0.47 mmol). Product **5a** (163 mg, 97%) was obtained as pink solid (m.p. 258.7–262.3 °C).
^{1}H NMR (300 MHz, $CDCl_3$): δ = 7.95 (bs, 1H), 7.48 (d, *J* = 6.2 Hz, 1H), 7.34 (d, *J* = 5.5 Hz, 1H), 7.22-7.07 (m, 2H), 7.02-6.96 (m, 4H), 6.16 (bs, 1H), 3.85 (dd, *J* = 3.8, 6.7 Hz, 1H), 3.64 (d, *J* = 16.6 Hz, 1H), 3.60 (s, 3H), 3.35 (dd, *J* = 5.2, 16.1 Hz, 1H), 3.23 (d, *J* = 16.6 Hz, 1H), 3.12 (dd, *J* = 3.8, 16.1 Hz, 1H), 2.29 (s, 3H), 1.89 (s, 3H) ppm. **^{13}C NMR APT** (75 MHz, $CDCl_3$): δ = 173.4 (C), 170.5 (C), 137.7 (C), 137.2 (C), 137.0 (C), 131.3 (C), 129.5 (2xCH), 128.2 (2xCH), 128.0 (C), 121.9 (CH), 119.7 (CH), 118.5 (CH), 111.0 (CH), 108.6 (C), 63.0 (C), 52.6 (CH), 44.4 (CH_3), 27.2 (CH_2), 25.8 (CH_2), 24.0 (CH_3), 21.3 (CH_3) ppm. **ESI (+)-MS**: m/z (%) = 399 (100) [M + Na]$^+$. $C_{23}H_{24}N_2O_3$ [376.46]: calcd. for C 73.38, H 6.43, N 7.34.; found C 73.45, H 6.32, N 7.48.

(±)-*cis*-Methyl 3-acetamido-2-(*p*-tolyl)-2,3,4,9-tetrahydro-1*H*-carbazole-3-carboxylate (5b)

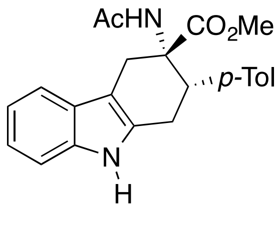

General procedure was followed using **3’a** (128 mg, 0.28 mmol) and K_2CO_3 (40.0 mg, 0.29 mmol). Product **5b** (90.0 mg, 87%) was obtained as pink solid (m.p. 262.4–269.2 °C).
^{1}H NMR (300 MHz, $CDCl_3$): δ = 7.89 (bs, 1H), 7.47 (d, *J* = 6.6 Hz, 1H), 7.33 (d, *J* = 5.1 Hz, 1H), 7.26-7.00 (m, 6H), 5.39 (bs, 1H), 4.08 (dd, *J* = 1.8, 5.1 Hz, 1H), 3.75 (dd, *J* = 7.7, 17.5 Hz, 1H), 3.60 (s, 3H), 3.58 (d, *J* = 15.6 Hz, 1H), 3.00 (dd, *J* = 1.8, 17.5 Hz, 1H), 2.78 (d, *J* = 15.6 Hz, 1H), 2.31 (s, 3H), 1.89 (s, 3H) ppm.

^{13}**C NMR APT** (75 MHz, $CDCl_3$): δ = 173.1 (C), 170.1 (C), 138.9 (C), 137.6 (C), 137.0 (C), 132.9 (C), 129.8 (2xCH), 128.7 (2xCH), 127.5 (C), 122.0 (CH), 119.8 (CH), 118.2 (CH), 111.1 (CH), 106.8 (C), 61.4 (C), 52.9 (CH_3), 42.6 (CH), 28.5 (CH_2), 28.0 (CH_2), 25.6 (CH_3), 21.4 (CH_3) ppm. **ESI (+)-MS**: m/z (%) = 399 (100) $[M + Na]^+$. $C_{23}H_{24}N_2O_3$ [376.45]: calcd. for C 73.38, H 6.43, N 7.34.

(±)-*trans*-Methyl 3-acetamido-2-methyl-2,3,4,9-tetrahydro-1*H*-carbazole-3-carboxylate (5c)

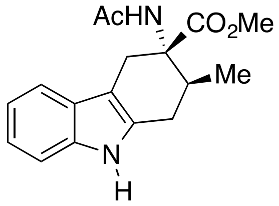

General procedure was followed using **3b** (226 mg, 0.61 mmol) and K_2CO_3 (89 mg, 0.64 mmol). Product **5c** (130 mg, 71%) was obtained as brown solid (m.p. 225.4–233.7 °C).
^{1}**H NMR** (200 MHz, $CDCl_3$): δ = 7.93 (bs, 1H), 7.48 (dd, *J* = 6.2, 1.8 Hz, 1H), 7.32-7.08 (m, 3H), 5.96 (bs, 1H), 3.77 (s, 3H), 3.30 (d, *J* = 8.4, 2H), 3.01 (dd, *J* = 5.9, 16.9 Hz, 1H), 2.73 (t, *J* = 6.5 Hz, 1H), 2.60 (d, *J* = 16.9 Hz, 1H), 1.88 (s, 3H), 1.07 (d, *J* = 7.1 Hz, 3H) ppm. ^{13}**C NMR APT** (50 MHz, $CDCl_3$): δ = 173.7 (C), 170.2 (C), 136.7 (C), 130.3 (C), 127.8 (C), 121.8 (CH), 119.6 (CH), 118.1 (CH), 111.0 (CH), 106.4 (C), 61.9 (C), 52.6 (CH_3), 33.4 (CH), 29.9 (CH_2), 25.3 (CH_2), 23.5 (CH_3), 16.4 (CH_3) ppm. **ESI(+)-MS**: m/z (%) = 301 (60) $[M + H]^+$; 323 (100) $[M + Na]^+$. $C_{17}H_{20}N_2O_3$ [300.36]: calcd. for C 67.98, H 6.71, N 9.33; C 68.25, H 6.88, N 9.21.

(±)-*trans*-Methyl 3-acetamido-2-cyclohexyl-2,3,4,9-tetrahydro-1*H*-carbazole-3-carboxylate (5d)

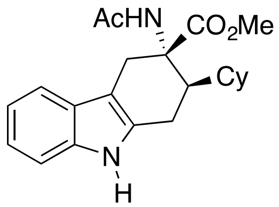

General procedure was followed using **3d** (520 mg, 1.18 mmol) and K_2CO_3 (171 mg, 1.24 mmol). Product **5d** (423 mg, 98%) was obtained as yellow solid (m.p. 254.4–261.6 °C).
^{1}**H NMR** (200 MHz, $CDCl_3$): δ = 7.80 (bs, 1H), 7.43 (dd, *J* = 0.7, 6.6 Hz, 1H), 7.30 (dd, *J* = 0.7, 7.0 Hz, 1H), 7.20-7.00 (m, 2H), 6.16 (bs, 1H), 3.75 (s, 3H), 3.60 (d, *J* = 16.5 Hz, 1H), 3.21 (d, *J* = 16.5 Hz, 1H), 2.88 (d, *J* = 5.1 Hz, 2H), 2.54 (t, *J* = 4.4 Hz, 1H), 1.91 (s, 3H), 2.10-0.60 (m, 11H) ppm. ^{13}**C NMR APT** (50 MHz, $CDCl_3$): δ = 174.3 (C), 169.8 (C), 136.8 (C), 133.0 (C), 128.1 (C), 120.7 (CH), 118.7 (CH), 117.7 (CH), 111.3 (CH), 106.1 (C), 62.4 (C), 52.4 (CH_3), 45.0 (CH), 38.4 (CH), 34.6 (CH_2), 28.5 (CH_2), 27.3 (CH_2), 26.8 (CH_2), 26.4 (CH_2), 25.8 (CH_2), 23.2 (CH_3), 20.5 (CH_2) ppm. **ESI(+)-MS**: m/z (%) = 369 (100) $[M + H]^+$. $C_{22}H_{28}N_2O_3$ [368.48]: calcd. for C 71.71, H 7.66, N 7.60; found C 71.43, H 7.78, N 7.52.

Preparation and characterization data for compounds (±)-6a–e

In a microwave vial the corresponding methyl 3-acetamido-2,3,4,9-tetrahydro-1*H*-carbazole-3-carboxylate **5a–d**, **3i** (1.00 equiv) was dissolved in HCl 37% (115 equiv). The vial was then introduced in a microwave (500W) and heated at 120 °C for 2 h. After that time solvent was removed under reduced pressure. The crude thus obtained was treated with propylene oxide (50 equiv) in ethanol at 80 °C for 1 h. Solvent was then removed and the crude purified by flash chromatography (SiO_2, ethyl acetate/methanol = 8:2) to yield the corresponding product **6** as a solid.

(±)-*trans*-3-Ammonio-2-(*p*-tolyl)-2,3,4,9-tetrahydro-1*H*-carbazole-3-carboxylate (6a)

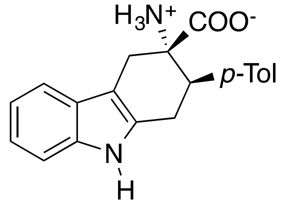

General procedure was followed using **5a** (250 mg, 0.66 mmol), HCl 37% (6.3 mL) and propylene oxide (2.27 mL, 32.3 mmol) to yield product **6a** (126.8 mg, 60%) as white solid (m.p. 221.8–222.3 °C).
^{1}H NMR (300 MHz, D_2O): δ = 7.54 (d, *J* = 7.8 Hz, 1H), 7.46 (d, *J* = 8.4 Hz, 1H), 7.20 (t, *J* = 7.2 Hz, 1H), 7.15-1.06 (m, 5H), 3.53 (t, *J* = 5.9 Hz, 1H), 3.33-3.20 (m, 3H), 3.01 (d, *J* = 16.2 Hz, 1H), 2.26 (s, 3H, CH_3) ppm. **^{13}C NMR APT** (75 MHz, D_2O): δ = 173.5 (C), 137.6 (C), 135.7 (C), 135.4 (C), 132.5 (C), 128.7 (2xCH), 127.6 (2xCH), 126.0 (C), 120.9 (CH), 118.6 (CH), 116.9 (CH), 110.6 (CH), 104.5 (C), 63.2 (C), 44.5 (CH), 26.4 (CH_2), 25.9 (CH_2), 19.4 (CH_3) ppm. **ESI(+)-MS**: m/z (%) = 321 (100) $[M + H]^+$. $C_{20}H_{20}N_2O_2$ [320.39]: calcd. for C 74.98, H 6.29, N 8.74; found: 75.11, H 6.33, N 8.68.

(±)-*cis*-3-Ammonio-2-(*p*-tolyl)-2,3,4,9-tetrahydro-1*H*-carbazole-3-carboxylate (6b)

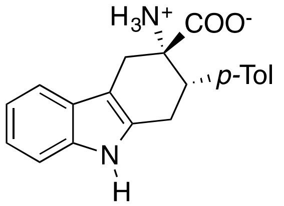

General procedure was followed using **5b** (90.4 mg, 0.24 mmol), HCl 37% (2.8 mL) and propylene oxide (0.82 mL, 11.8 mmol) to yield product **6b** (47.7 mg, 62%) as white solid (m.p. 220.2–221 °C).
^{1}H NMR (300 MHz, D_2O): δ = 7.45 (d, *J* = 7.3 Hz, 1H), 7.42 (d, *J* = 8.1 Hz, 1H), 7.25-7.13 (m, 5H), 7.10 (t, *J* = 7.2 Hz, 1H), 3.53 (dd, *J* = 6.5, 11.0 1H), 3.47 (d, *J* = 17.0 Hz, 1H), 3.16 (m, 2H), 2.94 (dd, *J* = 6.5, 11.0 Hz, 1H), 2.27 (s, 3H) ppm. **^{13}C NMR APT** (75 MHz, D_2O): δ = 173.3 (C), 138.0 (C), 135.8 (C), 133.4 (C), 131.7 (C), 129.0 (2xCH), 127.8 (2xCH), 125.7 (C), 121.1 (CH), 118.7 (CH), 116.9 (CH), 110.7 (CH), 102.7 (C), 63.7 (C), 43.2 (CH), 28.8 (CH_2), 24.0 (CH_2), 19.4 (CH_3) ppm.

(±)-trans-3-Ammonio-2-methyl-2,3,4,9-tetrahydro-1*H*-carbazole-3-carboxylate (6c)

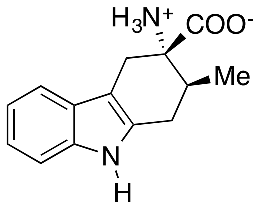

General procedure was followed using **5c** (106 mg, 0.35 mmol), HCl 37% (3.30 mL) and propylene oxide (1.20 mL, 17.2 mmol) to yield product **6c** (48.7 mg, 57%) as white solid (m.p. 217.7–218 °C).

^{1}H NMR (300 MHz, D_2O): δ = 7.37 (d, *J* = 7.3 Hz, 1H), 7.28 (d, *J* = 8.0, 1H), 7.15-7.06 (m, 2H), 3.48 (dd, *J* = 4.1, 11.6 Hz, 1H), 2.99 (m, 2H), 2.73 (dd, *J* = 3.75, 15.0 Hz, 1H), 2.53 (m, 1H), 1.07 (d, *J* = 6.4, 3H) ppm. **^{13}C NMR APT** (75 MHz, D_2O): δ = 173.7 (C), 136.8 (C), 132.2 (C), 126.9 (C), 122.0 (CH), 119.7 (CH), 117.9 (CH), 111.7 (CH), 103.4 (C), 71.2 (C) 34.2 (CH), 27.0 (CH_2), 26.0 (CH_2), 15.2 (CH_3) ppm. **ESI(+)-MS**: m/z (%) = 245 (100) $[M + H]^+$. $C_{14}H_{16}N_2O_2$ [244.29]: calcd. for C 68.83, H 6.60, N 11.47; C 68.54, H 6.72, N 11.61.

(±)- *trans*-3-Ammonio-2-cyclohexyl-2,3,4,9-tetrahydro-1*H*-carbazole-3-carboxylate (6d)

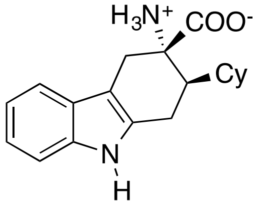

General procedure was followed using **5d** (50.0 mg, 0.13 mmol), HCl 37% (1.23 mL) and propylene oxide (0.50 mL, 7.14 mmol) to yield product **6d** (30.0 mg, 64%) as brown solid (m.p. 213.4–214 °C).

^{1}H NMR (300 MHz, D_2O): δ = 7.4 (d, *J* = 7.7 Hz, 1H), 7.31 (d, *J* = 8.1 Hz, 1H), 7.16 (dt, *J* = 1.1, 7.1 Hz, 1H), 7.10 (dt, *J* = 1.1, 8.1 Hz, 1H), 3.40 (d, *J* = 17.0 Hz, 1H), 3.30 (dd, *J* = 4.0, 18.0 Hz, 1H), 2.95 (d, *J* = 17.0 Hz, 1H), 2.88 (dd, *J* = 7.2, 18.0 Hz, 1H), 2.18 (m, 1H), 1.61-0.60 (m, 11H) ppm. **^{13}C NMR APT** (75 MHz, D_2O): δ = 175.1 (C), 135.6 (C), 132.8 (C), 126.0 (C), 120.7 (CH), 118.5 (CH), 116.7 (CH), 110.5 (CH), 103.7 (C), 63.3 (C), 44.0 (CH), 37.0 (CH), 33.0 (CH_2), 26.8 (CH_2), 26.4 (CH_2), 25.9 (CH_2), 25.4 (CH_2), 25.0 (CH_2), 19.0 (CH_2) ppm. **ESI(+)-MS**: m/z (%) = 313 (100) $[M + H]^+$; $C_{19}H_{24}N_2O_2$ [312.41]: calcd. for C 73.05, H 7.74, N 8.97; C 73.36, H 7.59, N 9.06.

(±)-3-Ammonio-2,3,4,9-tetrahydro-1*H*-carbazole-3-carboxylate (6e)

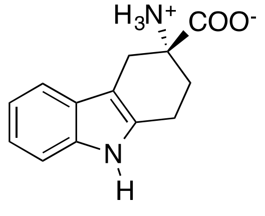

General procedure was followed using **3i** (440 mg, 1.53 mmol), HCl 37% (11 mL) and propylene oxide (4 mL, 57.1 mmol) to yield product **6e** (218 mg, 62%) as yellow solid (m.p. 219.4–220 °C).
^{1}H NMR (300 MHz, D_2O): δ = 7.38 (d, *J* = 7.0 Hz, 1H), 7.30 (d, *J* = 7.2 Hz, 1H), 7.15-6.94 (m, 2H), 3.40 (d, *J* = 17.0 Hz, 1H), 3.20 (d, *J* = 16.5 Hz, 1H), 2.91 (d, *J* = 16.5 Hz, 1H), 2.77-2.53 (m, 2H), 2.40-2.15 (m, 2H) ppm. **^{13}C NMR APT** (75 MHz, D_2O): δ = 177.1 (C), 136.5 (C), 132.6 (C), 126.9 (C), 121.8 (CH), 119.4 (CH), 117.7 (CH), 111.5 (CH), 104.2 (C), 61.9 (C), 28.8 (CH_2), 28.7 (CH_2), 18.3 (CH_2) ppm. **ESI(-)-MS**: m/z (%) = 229 (100) [M - H]$^{+}$; $C_{13}H_{14}N_2O_2$ [230.27]: calcd. for C 67.81, H 6.13, N 12.17; found C 67.69, H 6.28, N 12.02.

2D NMR EXPERIMENTS

Representative NOESY experiments performed on compounds 3a and 3’a

For clarity chemical shift of proper protons, assigned via COSY and HSQC experiments, are reported.
The regiochemistry of both DA adducts was usefully assigned on the basis of nOe interactions between H5 and the hydrogens at C4, see figure A and A’ for **3a** and **3’a**, respectively. The *exo*/*endo* geometries were assigned on the basis of diagnostic nOe interactions as reported in figures B and B’. Due to signals overlapping the most useful interactions were detected, for both compounds, between the NH group and the hydrogens in *cis* to this group on carbons 1, 2 and 4. NOE interactions between the hydrogens bonded at C1, C2 and C4 are difficult to detect due to signals overlapping. The sole information that can be unambiguously noticed refers to the benzylic proton at C2. In both **3a** and **3’a**, the hydrogen at C2 do not interact with the *trans* hydrogen at C4, figure C and figure C’, respectively.

Compound 3a

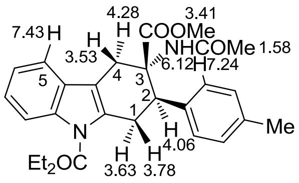

(±)-3a, 2,3-*trans*

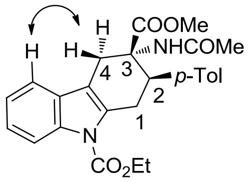

Regiochemistry **3a**

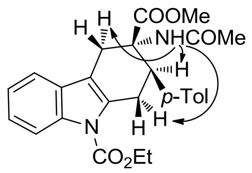

Stereochemistry **3a**
Diagnostic NOE interactions

NOESY experiements

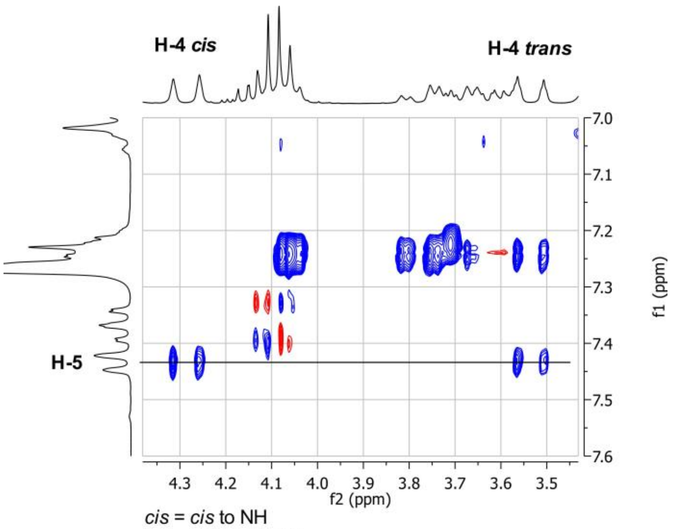

cis = *cis* to NH
trans = *trans* to NH

FIGURE A

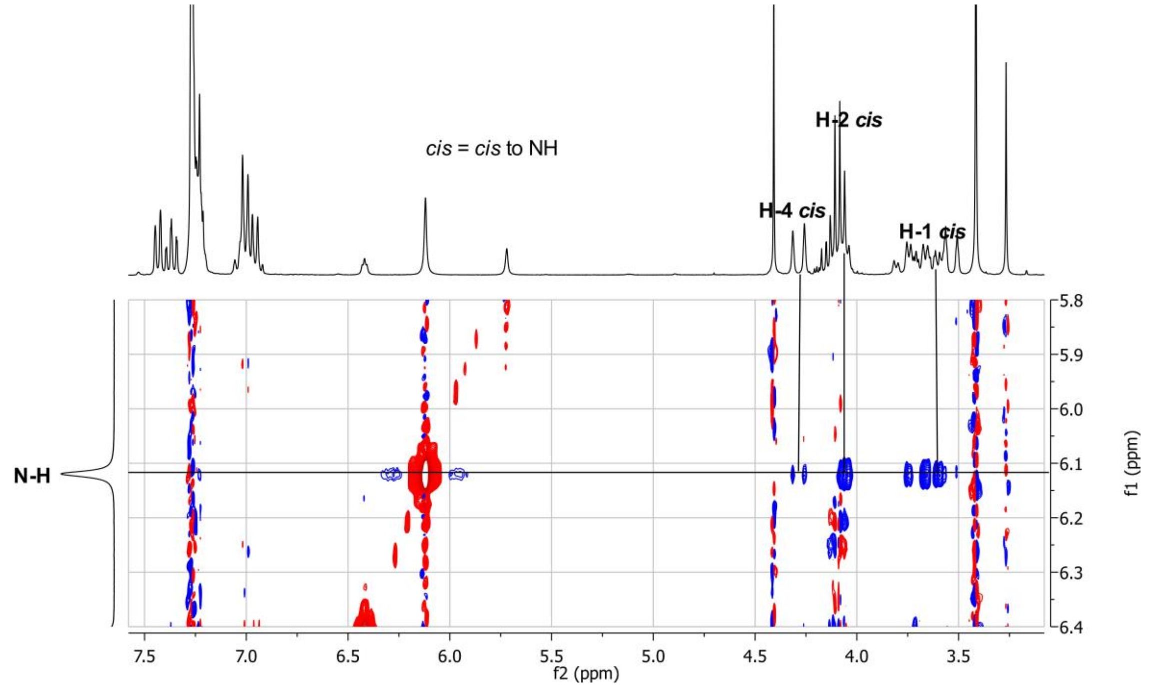

FIGURE B

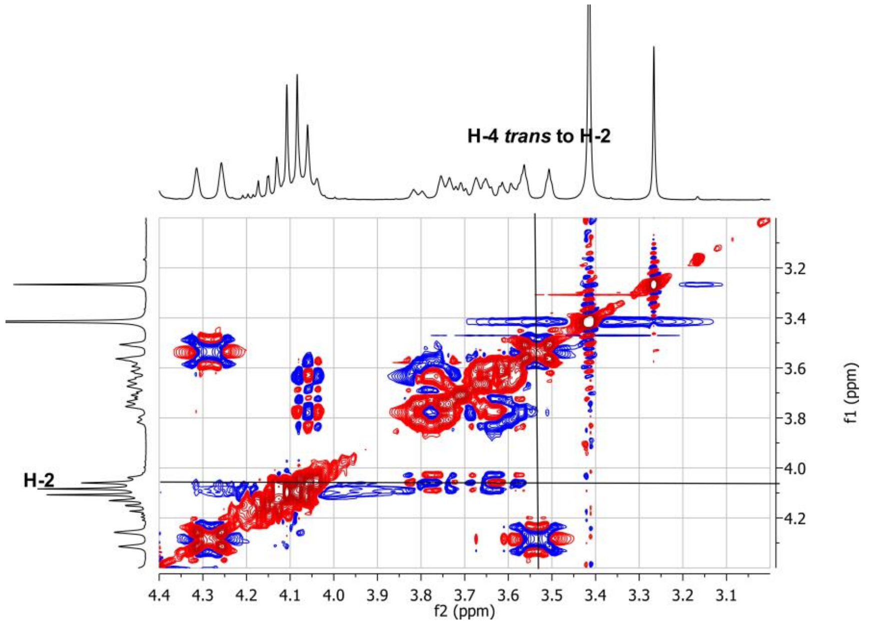

FIGURE C

Compound 3'a

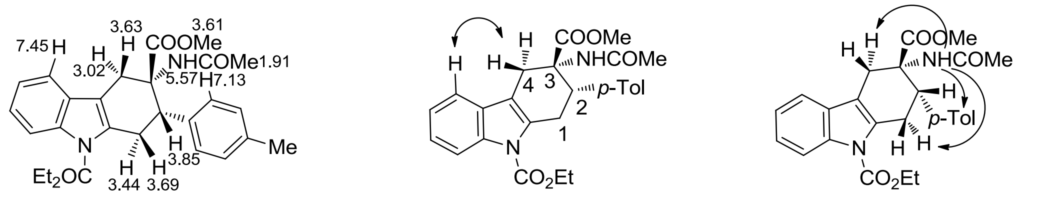

(±)-3'a, 2,3-*cis*

Regiochemistry **3'a**

Stereochemistry **3'a**
Diagnostic NOE interactions

NOESY experiements

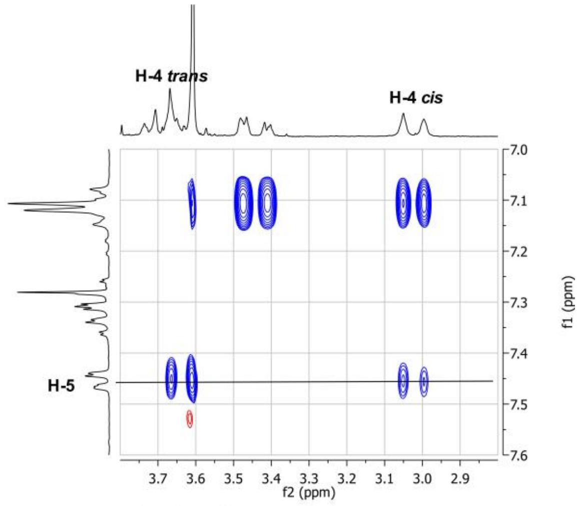

cis = *cis* to NH
trans = *trans* to NH

FIGURE A'

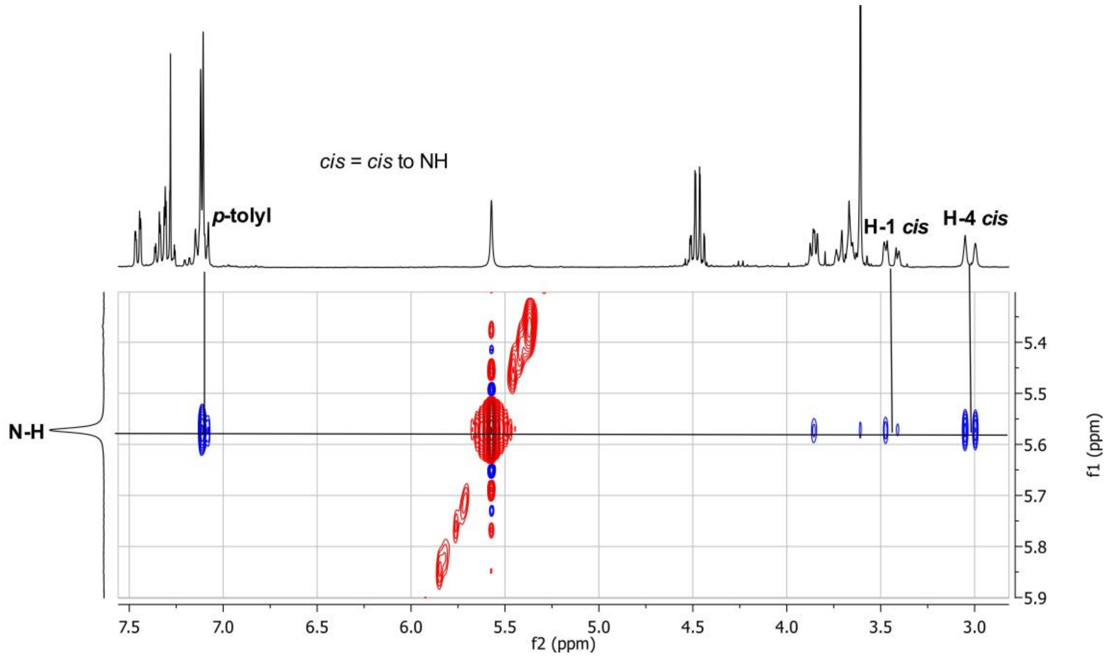

FIGURE B'

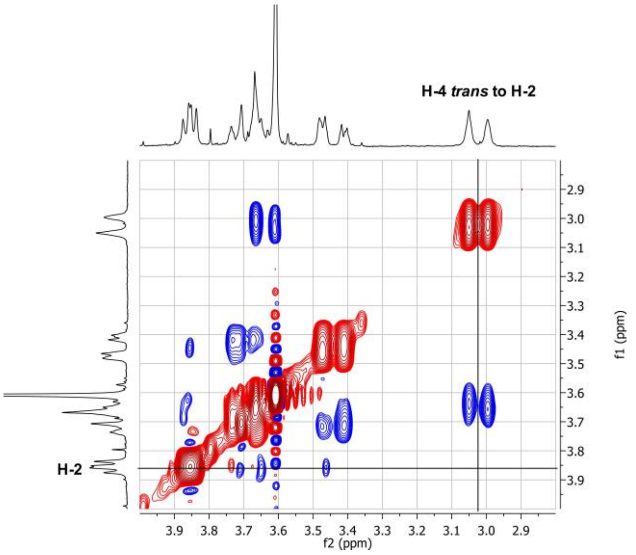

FIGURE C'

Representative NMR experiments (^{1}H, ^{13}C, COSY, HSQC) performed on compounds 6a and 6b

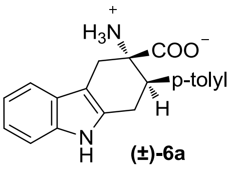

(±)-6a

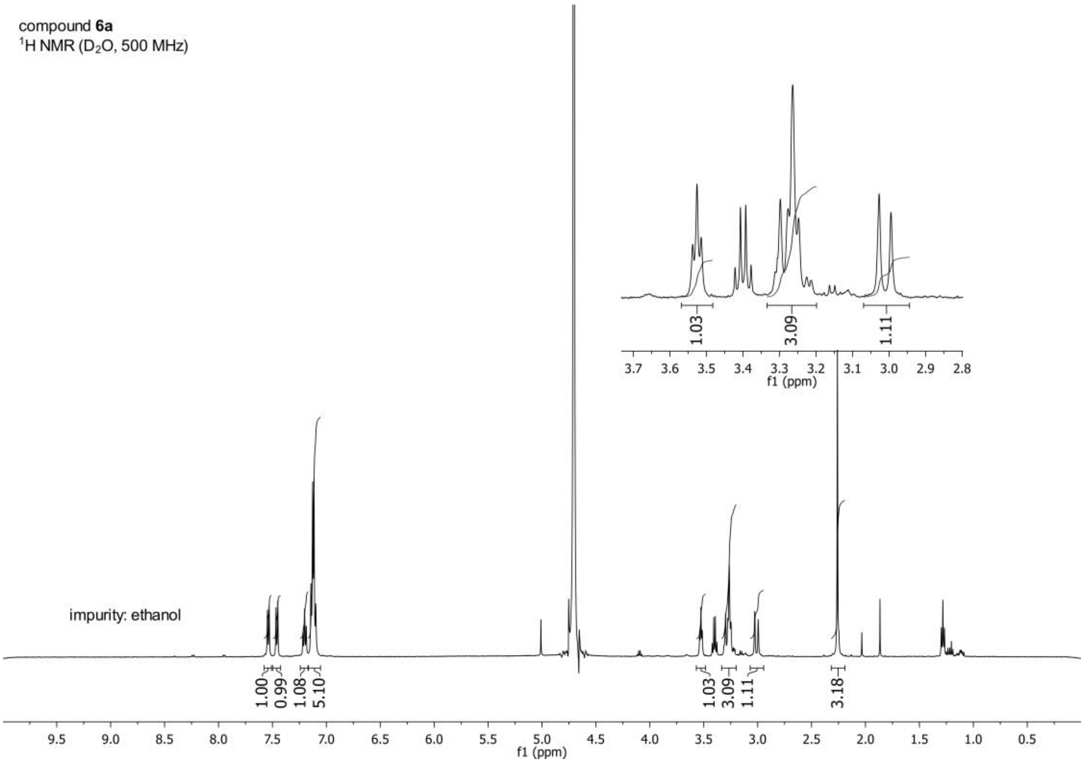

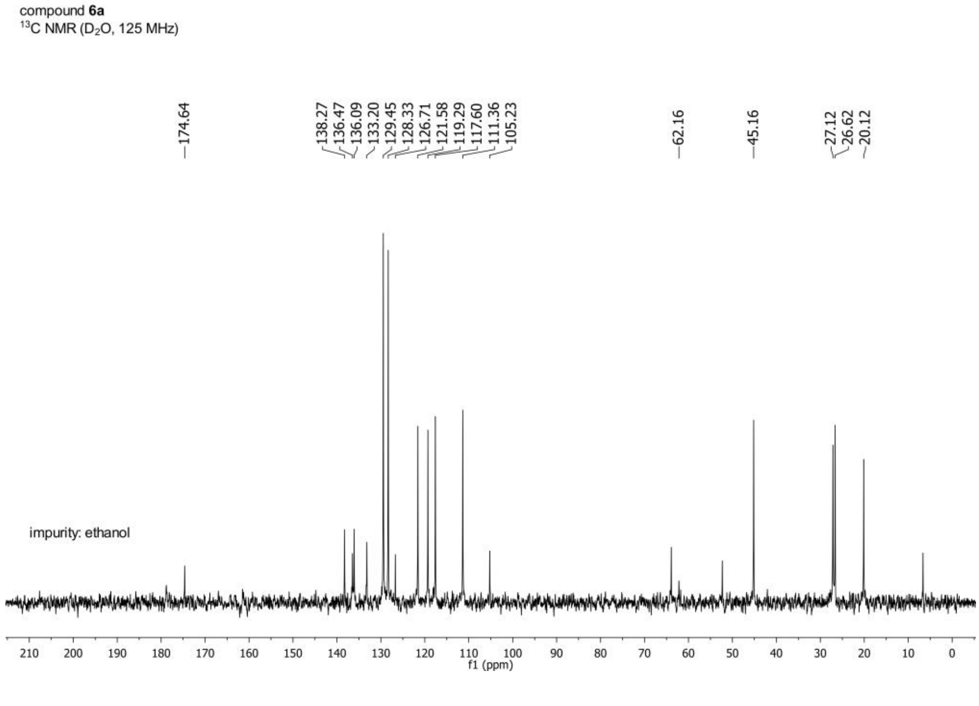

compound **6a**
COSY NMR (D_2O, 500 MHz)

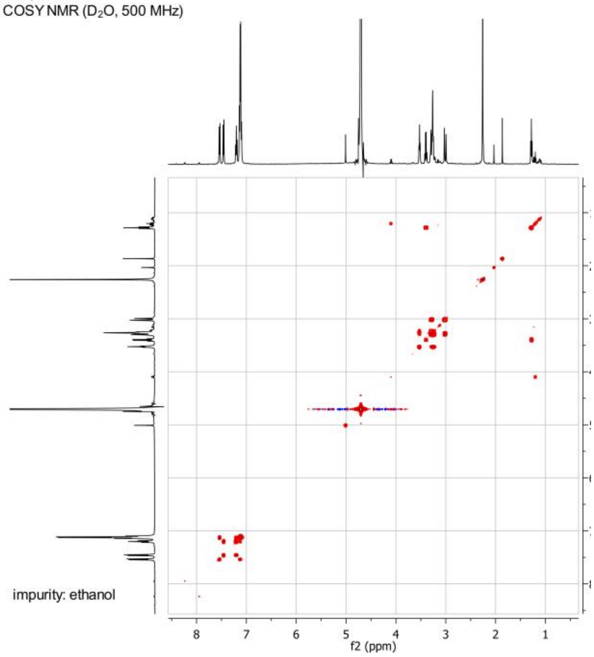

compound **6a**
HSQC NMR (D_2O, 500 MHz)

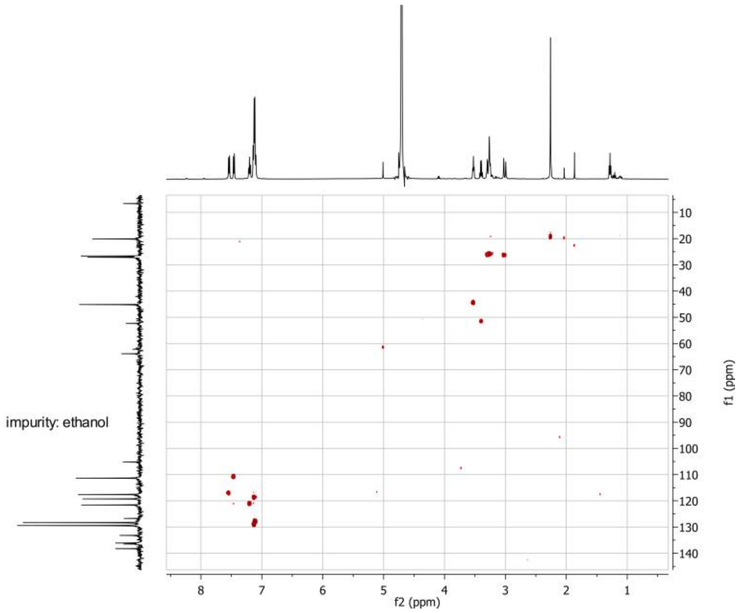

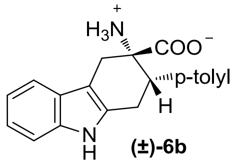
H3N
COO
p-tolyl
H
N
H

(±)-6b

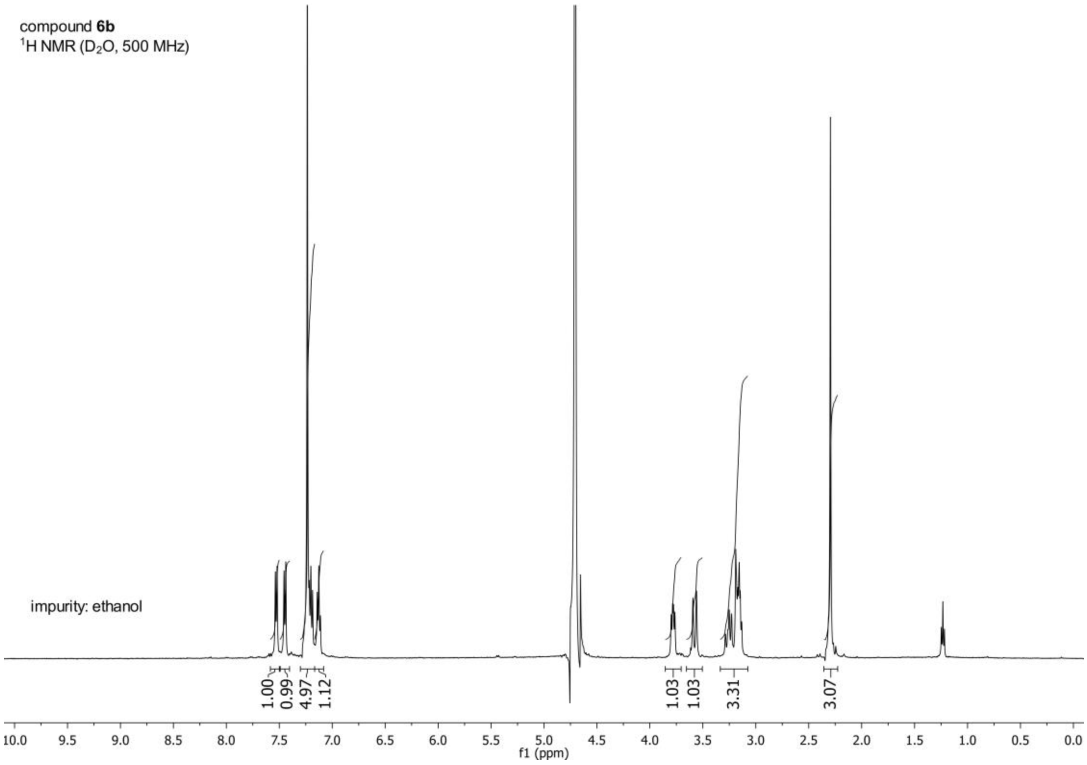
compound **6b**
1H NMR (D2O, 500 MHz)
impurity: ethanol
1.00
0.99
4.97
1.12
1.03
1.03
3.31
3.07
10.0 9.5 9.0 8.5 8.0 7.5 7.0 6.5 6.0 5.5 5.0 4.5 4.0 3.5 3.0 2.5 2.0 1.5 1.0 0.5 0.0
f1 (ppm)

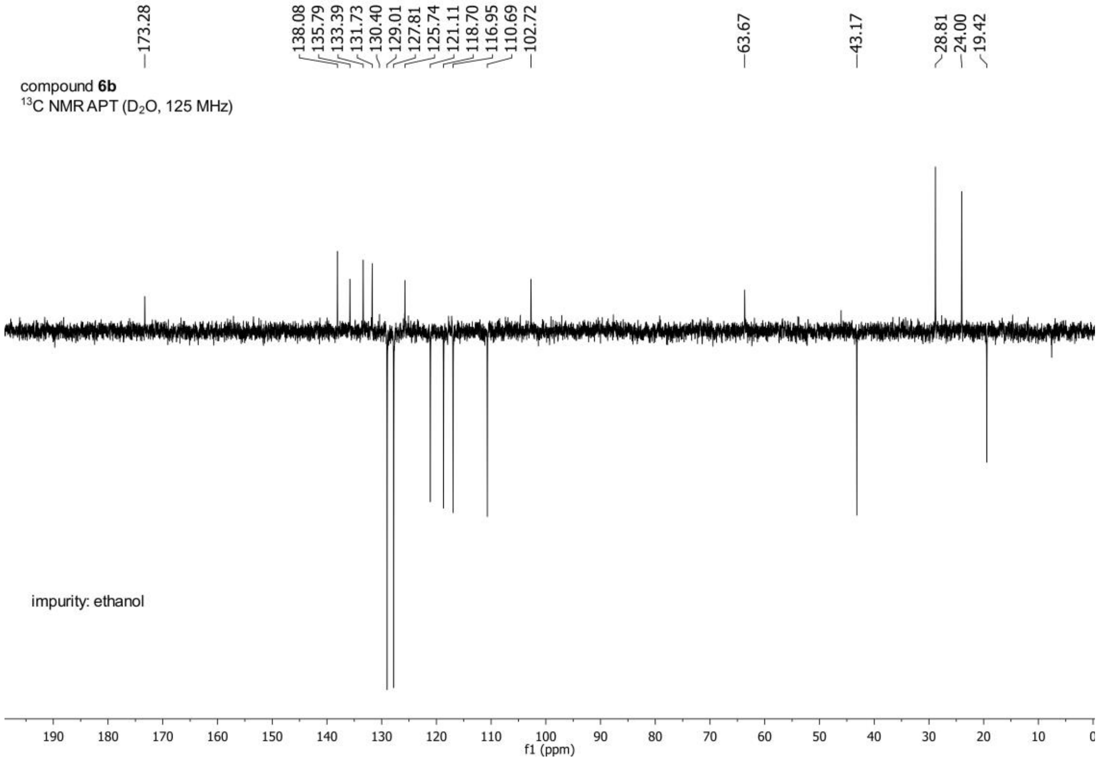
173.28
138.08
135.79
133.39
131.73
130.40
129.01
127.81
125.74
121.11
118.70
116.95
110.69
102.72
63.67
43.17
28.81
24.00
19.42
compound **6b**
13C NMR APT (D2O, 125 MHz)
impurity: ethanol
190 180 170 160 150 140 130 120 110 100 90 80 70 60 50 40 30 20 10 0
f1 (ppm)

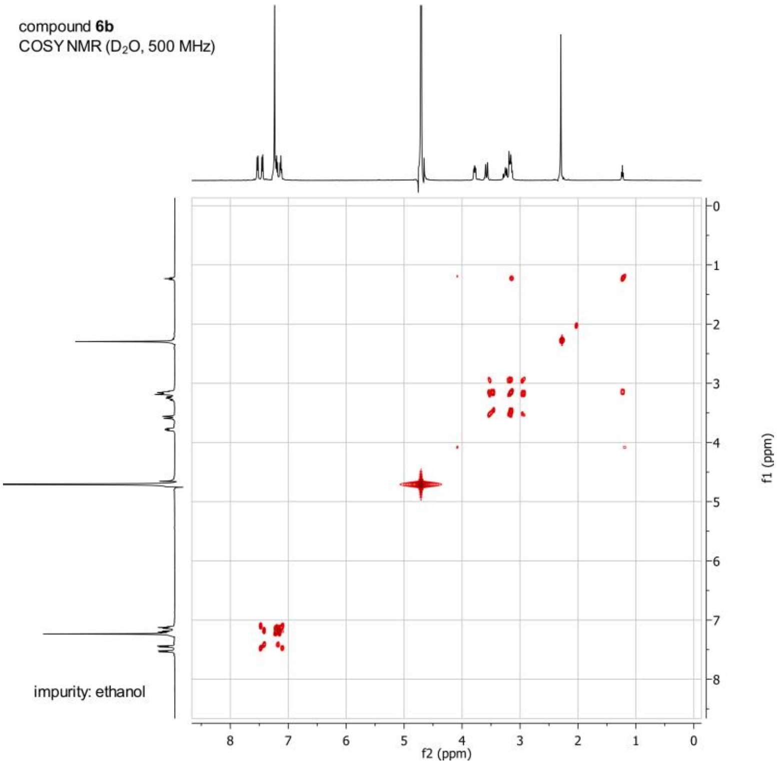

compound 6b
COSY NMR (D_2O, 500 MHz)
impurity: ethanol
f2 (ppm)
f1 (ppm)

References

[1] a) Rossi, E.; Abbiati, G.; Canevari, V.; Celentano, G. *Synthesis* **2006**, 299-304; b) Abbiati, G.; Canevari, V.; Facoetti, D.; Rossi, E. *Eur. J. Org. Chem.* **2007**, 517-525; c) Pirovano, V.; Decataldo, L.; Vicente, R.; Rossi, E. *Chem. Commun.* **2013**, *49*, 3594-3596.

[2] Waser, J.; Gaspar, B.; Nambu, H.; Carreira, E. M. *J. Am. Chem. Soc.* **2006**, *128*, 11693-11712.

[3] Crestey, F.; Collot, V.; Stiebing, S.; Rault, S. *Synthesis* **2006**, 3506-3014.
